# Prevalence and Variability of Clinical Manifestations of Dengue in Peru: A Systematic Review and Meta-Analysis of Observational Studies

**DOI:** 10.3390/v18070732

**Published:** 2026-07-02

**Authors:** Darwin A. León-Figueroa, Edwin Aguirre-Milachay, Dorothy Luisa Meléndez Morote, Miguel Villegas-Chiroque, Víctor J. Vera-Ponce, Oriana Rivera-Lozada, Mario J. Valladares-Garrido

**Affiliations:** 1 Facultad de Medicina Humana, Universidad de San Martín de Porres, Chiclayo 15011, Peru; darwin_leon@usmp.pe; 2 EpiHealth Research Center for Epidemiology and Public Health, Lima 15001, Peru; 3 Hospital Nacional Almanzor Aguinaga Asenjo, EsSalud, Chiclayo 14001, Peru; edwinh.aguirre@gmail.com; 4 Programa de Odontología, Universidad de San Martín de Porres, Chiclayo 15011, Peru; dmelendezm@usmp.pe; 5 Escuela de Medicina Humana, Universidad Señor de Sipán, Chiclayo 14001, Peru; mvillegasch@uss.edu.pe (M.V.-C.); riveraoriana@uss.edu.pe (O.R.-L.); 6 Facultad de Medicina (FAMED), Universidad Nacional Toribio Rodríguez de Mendoza de Amazonas, Chachapoyas 01001, Peru; victor.vera@untrm.edu.pe; 7 Oficina de Inteligencia Sanitaria, Red Prestacional EsSalud Lambayeque, Chiclayo 14001, Peru

**Keywords:** clinical manifestations, dengue, *Aedes aegypti*, Peru, meta-analysis

## Abstract

Dengue remains a major public health challenge in Peru, where recurrent outbreaks show marked variation in clinical presentation. This systematic review and meta-analysis synthesized available evidence to quantify the frequency and variability of dengue manifestations in Peruvian patients and to identify clinically relevant patterns for early recognition. We systematically searched PubMed, Scopus, Embase, Web of Science, ScienceDirect, Google Scholar, Virtual Health Library, and Scielo for observational studies published between 1993 and January 2025. Two reviewers independently selected studies, extracted data, and assessed methodological quality. Pooled prevalence estimates with 95% confidence intervals (CIs) were calculated using random-effects models. Twenty-eight studies including 4418 patients were analyzed. The most frequent manifestations were fever (95%; 95% CI: 90–98%), headache (86%; 95% CI: 80–91%), malaise (82%; 95% CI: 71–91%), myalgia (69%; 95% CI: 58–79%), arthralgia (64%; 95% CI: 56–73%), and retro-orbital pain (56%; 95% CI: 47–66%). Gastrointestinal symptoms were also common, including nausea/vomiting (40%; 95% CI: 33–48%) and abdominal pain (33%; 95% CI: 21–45%), whereas hemorrhagic and severe manifestations were less frequent, such as hematemesis (6%; 95% CI: 2–10%), petechiae (6%; 95% CI: 2–10%), jaundice (3%; 95% CI: 1–7%), and melena (1%; 95% CI: 0–6%). Heterogeneity was high across most outcomes (I^2^ generally >90%), suggesting substantial between-study variability. This heterogeneity is likely related to differences in geographic region, outbreak period, circulating serotypes, diagnostic methods, and case severity definitions across studies. These findings highlight a consistent core symptom profile of dengue in Peru while also demonstrating important clinical variability. This information may support earlier clinical suspicion, triage, and surveillance in endemic settings. However, pooled estimates should be interpreted cautiously given the high heterogeneity, moderate methodological rigor of included studies, and lack of individual-level data. Future analyses stratified by region, study period, and diagnostic method are needed to generate more clinically precise estimates.

## 1. Introduction

Dengue is an infectious disease caused by dengue virus (DENV), a single-stranded RNA virus belonging to the family Flaviviridae and genus Flavivirus [1]. This virus has four known serotypes (DENV 1, DENV 2, DENV 3, and DENV 4) [2,3]. Transmission of this virus occurs through the bite of female mosquitoes of the genus *Aedes* spp., mainly by *Ae. aegypti* and occasionally by *Ae. albopictus* [4,5]. *Ae. aegypti* was first described by Linnaeus in 1762, while *Ae. albopictus* was described by Skuse in 1894 [6].

According to the World Health Organization (WHO), dengue is classified into two main categories: dengue (with/without warning signs) and severe dengue [7]. According to the U.S. Centers for Disease Control and Prevention, common symptoms of dengue include fever, headache, myalgia, arthralgia, retroocular pain, nausea, vomiting, and rash [8]. These symptoms usually last 2 to 7 days, and most people recover in about a week [8].

Dengue has affected over 14.6 million people worldwide in 2024, with a particularly significant impact in tropical and subtropical regions, making it the year with the highest number of recorded cases to date [7]. In 2025, the Pan American Health Organization (PAHO) reported over 4.4 million suspected cases of dengue and more than 1.6 million confirmed cases in the Americas region [9]. By 2026, as of epidemiological week 9, more than 359,000 suspected cases and over 76,000 confirmed cases were reported. In terms of country-specific impact, Brazil is the most affected, with over 280,000 reported cases, followed by Colombia with more than 20,000, Bolivia with over 14,000, Mexico with over 10,000, and Peru with over 8000 cases [10].

In recent years, Peru has experienced periodic outbreaks of dengue fever, with changes in incidence and a variety of clinical manifestations observed, ranging from asymptomatic patients to severe dengue cases [11]. According to data provided by the dengue situational room of the Peruvian National Center for Epidemiology, Prevention, and Disease Control (CDC), more than 895 thousand cases of dengue were recorded, with more than 1200 deaths, from 2014 to week 9 of 2026 [12]. The increase in dengue in Peru is mainly influenced by the meteorological conditions of the Coastal El Niño phenomenon, a lack of a solid and efficient health care system, political problems, and rapid unplanned urbanization [13,14,15,16]. This has had a considerable impact on public health in the country.

Recognizing specific clinical manifestations is essential for early detection of severe dengue, enabling timely diagnosis and the application of more effective clinical interventions. Furthermore, understanding the factors influencing disease progression can inform prevention strategies and improve healthcare management protocols, ultimately reducing dengue-related morbidity and mortality in Peru [11]. Although previous studies from Latin America and other endemic settings have described the clinical spectrum of dengue, their findings are not directly generalizable to Peru because of differences in circulating serotypes, outbreak intensity, ecological conditions, and access to healthcare. In addition, the available evidence in Peru remains fragmented across studies conducted in different regions and time periods, with substantial variation in reported clinical manifestations. To our knowledge, no previous systematic review and meta-analysis has specifically synthesized Peruvian observational studies to provide pooled estimates of the prevalence of dengue clinical manifestations at the national level. This gap is particularly relevant in the context of the recent increase in dengue cases in Peru, which underscores the need for an updated country-specific synthesis to support early clinical recognition, risk stratification, and public health decision-making [17,18].

Therefore, this study aims to determine the prevalence of clinical manifestations of dengue in Peruvian patients, thereby addressing an important evidence gap in the national literature and providing a detailed and updated overview to enhance early diagnosis and clinical management. The findings are expected to support public health and clinical strategies for better prevention, diagnosis, and treatment of dengue. Additionally, the information obtained could serve as a valuable resource for future research and health policy decision-making related to dengue in the region [19].

## 2. Materials and Methods

### 2.1. Protocol and Registration

The present investigation was carried out following the guidelines of Preferred Reporting Items for Systematic Reviews and Meta-Analysis (PRISMA) (Table S1) [20], as well as a protocol registered in the Prospective International Registry of Systematic Reviews (PROSPERO) with identification number CRD42024541811. Additionally, systematic reviews and meta-analyses conducted in Peru on infectious diseases were reviewed and followed, allowing for adherence to a rigorous methodology and a better understanding of the clinical variables [21,22,23]. This systematic review was carried out in several stages: development of a protocol, identification of key words, database searches for article selection, definition of inclusion and exclusion criteria, critical appraisal of studies, data selection and analysis, and presentation and interpretation of results.

### 2.2. Eligibility Criteria

This review included observational studies, such as retrospective, prospective, cohort, case–control, and cross-sectional studies, which investigated the prevalence of clinical manifestations in Peruvian patients diagnosed with dengue. Only those cases diagnosed either through laboratory tests—such as IgM ELISA (enzyme-linked immunosorbent assay), IgG ELISA, NS1 tests detecting the non-structural protein 1 (NS1), and RT-PCR (reverse transcriptase polymerase chain reaction)—or based on clinical criteria (a set of observed characteristics, signs, and symptoms) as per national and international guidelines, were included in the analyses. Studies that did not meet the established criteria, such as case reports, editorials, letters to the editor, randomized clinical trials, conference abstracts, and narrative or systematic reviews, were excluded. Although randomized clinical trials may provide useful baseline data on clinical manifestations, they were excluded because this review focused on observational studies designed to estimate symptom prevalence in the general population. Future studies may consider RCTs to explore baseline characteristics and clinical progression in greater detail. Likewise, patients with coinfections of dengue and other diseases (such as COVID-19, Zika, and chikungunya) were excluded.

### 2.3. Information Sources and Search Strategy

Eight databases were searched, including PubMed, Scopus, Embase, Web of Science, ScienceDirect, Google Scholar, Virtual Health Library (VHL), and Scielo, until 12 January 2025, with no language or development period restrictions. The following MeSH (Medical Subject Headings) terms were used in the search: “dengue”, “dengue fever”, and “Peru”, combined using the Boolean operators AND and OR. The search strategy was independently validated by two authors (V.J.V-P and M.J.V-G) and is detailed in Table S2. In addition, other search methods were carried out, such as reviewing bibliographic studies, consulting article references, and exploring publications in Peruvian specialized journals on infectious and communicable diseases. However, the potential studies identified were within the scope of the search strategy employed.

### 2.4. Study Selection

The results of the search strategy were stored in EndNote version X9 software (Clarivate [formerly part of Thomson Reuters], New York, NY, USA). Subsequently, duplicate articles, as well as repeated titles and abstracts, were removed. Next, the titles and abstracts of the articles were independently reviewed (D.L.M.M., M.V.-C., and O.R-L) to select those that met the inclusion criteria. Finally, a thorough review of the full articles was performed to determine their compliance with the inclusion criteria. Any discrepancies identified were resolved by mutual agreement.

### 2.5. Outcomes

The main objective is to establish the prevalence of clinical manifestations among Peruvian patients diagnosed with dengue.

### 2.6. Quality Assessment

The JBI-MAStARI (Joanna Briggs Institute Meta-Analysis of Statistics Assessment and Review Instrument) tool was used to assess the quality and risk of bias of the articles included in the meta-analysis. The assessment considered several elements, including study context, outcomes and explanatory variables, specific inclusion criteria, measurement methods used, a detailed description of the topic, and rigorous statistical analysis. The quality of the studies was classified as high (≥7 points), moderate (4 to 6 points), or low (<4 points) based on their scores [24] (Table S3).

Additionally, a critical analysis of the studies was conducted to evaluate the methodological rigor and consistency of the reported outcomes [25]. This involved assessing the appropriateness of study designs in relation to the research question, the validity of outcome measures, the handling of confounding variables, and the applicability of findings across diverse clinical contexts. Studies with inconsistent or poorly defined outcomes, inadequate control of confounders, or methodological limitations that could compromise the validity of frequency and association measures were carefully examined and discussed. This process ensured that the heterogeneity among studies did not hinder the feasibility and validity of combining frequency and association measures in the meta-analysis [26,27].

### 2.7. Data Collection Process and Data Items

The data from the articles was compiled into an Excel spreadsheet (Table S4). Two authors (D.A.L-F and E.A-M) manually and independently extracted a series of data including: author, year of publication, study design, study sample, region of development, sex (male and female), age of participants, dengue subtypes, study period, sample characteristics, types of dengue diagnostic techniques (RT-PCR, ELISA, and immunofluorescent assay) or through clinical criteria, types of biomarkers detected (IgM and IgG), final outcome (recovered and deceased), data collection methods, and clinical manifestations of dengue patients (chills, fever, headache, retro-orbital pain, myalgia, arthralgia, malaise, nausea/vomiting, abdominal pain, diarrhea, hematemesis, rash, jaundice, lumbago, cough, sore throat, melena, petechiae, and ecchymosis).

The selection of clinical manifestations and diagnostic methods was based on the guidelines provided by the WHO for the diagnosis and management of dengue, as well as on the recommendations of the Peruvian Ministry of Health. Data were extracted strictly for cases where the diagnosis of dengue had been previously established in the primary studies, either by laboratory techniques (such as RT-PCR, ELISA, and immunofluorescent assay) or based on standardized clinical criteria, as outlined by national and international health guidelines. Criteria for the selection of signs and symptoms included their frequency, clinical relevance, and usefulness in differentiating between dengue and other febrile illnesses [28,29].

At a meeting, the extractions performed by the two independent authors (D.A.L-F and E.A-M) were compared, and discrepancies were resolved by mutual agreement. Subsequently, to ensure the accuracy and quality of the extracted data, a rigorous review and verification process was carried out by a third independent investigator (M.J.V-G).

### 2.8. Data Analysis

A prevalence meta-analysis (proportions) was performed using R software version 4.2.3 (https://www.r-project.org/, accessed on 20 March 2025) (Table S5). An inverse variance-weighted random effects model was used to calculate the pooled prevalence of clinical manifestations in Peruvian patients diagnosed with dengue. The Cochrane Q statistic was applied to assess between-study variability. Heterogeneity between studies was assessed using the Inconsistency Index (I^2^), classifying heterogeneity as low if it was less than 25%, moderate if it was between 25% and 50%, and high if it exceeded 75% [30,31].

Prior to data synthesis, the relevance and comparability of the primary studies were thoroughly evaluated to ensure consistency with the research question. This assessment considered the alignment of study objectives, population characteristics, diagnostic methods for dengue, and definitions of clinical manifestations. Only studies with sufficient methodological and clinical similarity were included in the meta-analysis, ensuring that the combination of results was both appropriate and scientifically valid [26].

To assess possible publication bias, two methods were used: visual inspection of the funnel plot and Egger’s test. The latter was applied only to clinical manifestations with at least 10 studies included in the meta-analysis, since the test has less power to detect real asymmetry when fewer studies are included. This threshold of 10 studies for Egger’s test is explicitly stated in the Results section. Bias was considered to exist in the results when the resulting *p*-value was less than 0.05 [32].

The results of the investigation were presented in tables and descriptive graphs. The combined prevalence of clinical manifestations in Peruvian patients diagnosed with dengue was graphically illustrated using a forest plot, including 95% confidence intervals for a more accurate presentation of the data.

## 3. Results

### 3.1. Study Selection

**Figure 1:**
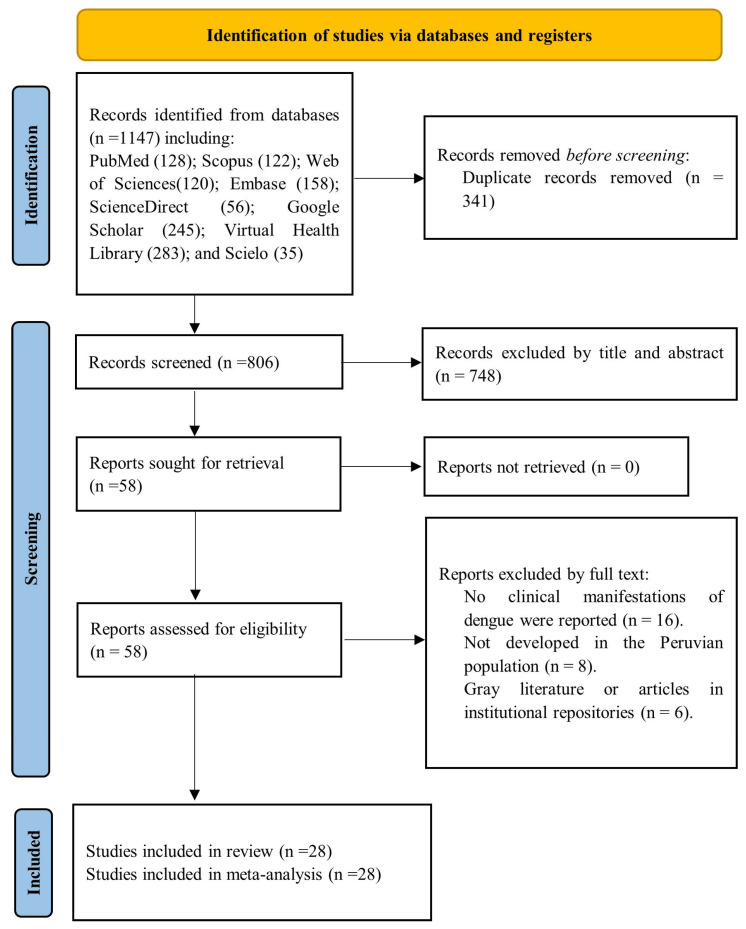
Study selection process based on the PRISMA flowchart.

A total of 1147 studies were retrieved through database searches. After eliminating duplicate articles (*n* = 341), the authors reviewed the remaining 806 through titles and abstracts. Subsequently, 58 articles were evaluated in full text, of which 28 met the inclusion criteria for the systematic review and meta-analysis [33,34,35,36,37,38,39,40,41,42,43,44,45,46,47,48,49,50,51,52,53,54,55,56,57,58,59,60]. The selection process is detailed in the PRISMA flow chart, shown in Figure 1.

### 3.2. Characteristics of the Included Studies

**Figure 2:**
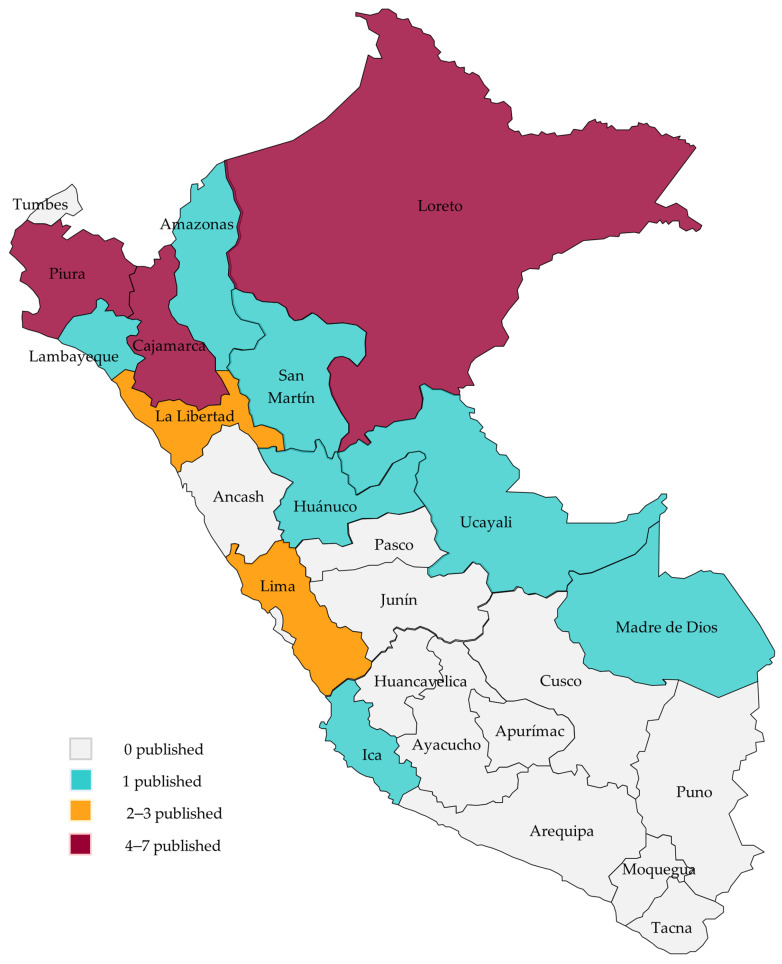
Geographic visualization of the regions where the studies were carried out in Peruvian patients with dengue: Piura (*n* = 6) [34,40,50,55,57,59], Loreto (*n* = 7) [37,46,47,52,53,54,60], Lima (*n* = 2) [39,58], La Libertad (*n* = 2) [41,45], Cajamarca (*n* = 4) [36,42,44,56], Lambayeque (*n* = 1) [49], Huánuco (*n* = 1) [48], Ica (*n* = 1) [43], Ucayali (*n* = 1) [33], Amazonas (*n* = 1) [35], Madre de Dios (*n* = 1) [51], and San Martín (*n* = 1) [38].

The analysis was based on a review of 28 observational articles published between 1993 and 2024 that investigated the prevalence of clinical manifestations in Peruvian patients diagnosed with dengue (Table 1) [33,34,35,36,37,38,39,40,41,42,43,44,45,46,47,48,49,50,51,52,53,54,55,56,57,58,59,60]. A total of 4418 patients with dengue were included, of whom, with available data, approximately 40.6% (1794) were men and 40.4% (1786) were women. The most frequent age group was 20 to 40 years old [33,34,35,36,37,38,39,40,41,42,43,44,45,46,47,48,49,50,51,52,53,54,55,56,57,58,59,60]. The studies were conducted in 12 regions of Peru, with a main focus on Piura and Loreto (Figure 2).

The most prevalent serotype was DENV2 (29.56%; 301/1018), followed by DENV3 (26.52%; 270/1018), while DENV1 and DENV4 have lower prevalences, 16.50% (168/1018) and 14.93% (152/1018), respectively [35,42,44,46,50,51,52,54,55,58]. Patients with dengue were diagnosed by screening tests, including ELISA for the nonstructural glycoprotein NS1, as well as detection of IgM or IgG antibodies and RT-PCR. Most patients recovered, although 56 deaths were reported (Table 1) [33,34,35,36,37,38,39,40,41,42,43,44,45,46,47,48,49,50,51,52,53,54,55,56,57,58,59,60].

### 3.3. Quality of the Included Studies and Publication Bias

Study quality was assessed using the JBI critical appraisal tools designed specifically for observational research. All studies included in the analysis were found to demonstrate a moderate level of quality, as indicated in Table S3 [33,34,35,36,37,38,39,40,41,42,43,44,45,46,47,48,49,50,51,52,53,54,55,56,57,58,59,60]. The absence of high-quality studies was mainly due to common methodological limitations in observational research, such as incomplete control of confounding variables, variability in outcome measurement, and limited reporting of methodological procedures. Despite these limitations, the studies met the inclusion criteria and provided sufficient methodological rigor to estimate frequency and association measures [61]. Therefore, although the overall quality was moderate, the consistency of findings across studies supports the reliability of the pooled estimates, and the results should be interpreted with appropriate caution.

In the analyses aimed at evaluating the clinical manifestations of Peruvian patients with dengue, it was observed that when Egger’s test was applied to assess publication bias, the following results were obtained: fever (*p* = 0.8505) [34,35,36,37,38,40,41,42,43,44,45,46,47,48,49,50,51,52,54,55,56,57,58,59,60], headache (*p* = 0.3265) [33,34,35,36,37,38,39,40,41,42,43,44,45,46,47,48,49,50,51,52,54,55,56,57,58,59,60], retro-orbital pain (*p* = 0.0980) [33,34,35,36,38,39,40,41,42,43,44,45,46,48,49,50,51,52,55,56,57,58,59,60], myalgia (*p* = 0.4298) [33,34,35,36,38,39,40,41,42,43,44,45,46,47,48,49,50,51,52,54,55,56,58,59,60], general malaise (*p* = 0.6333) [34,36,46,47,51,56,57,58,59,60], nausea/vomiting (*p* = 0.0530) [33,34,35,36,37,38,39,40,41,42,43,44,45,46,48,50,51,52,53,54,55,57,58,59,60], abdominal pain (*p* = 0.0977) [33,34,38,39,40,41,42,43,44,46,48,49,50,51,52,53,54,55,56,57,58,59], diarrhea (*p* = 0.3986) [33,34,37,38,39,42,44,46,51,52,53,54,55,57,58], rash (*p* = 0.5834) [33,34,35,36,37,38,39,40,41,42,44,48,49,50,51,52,54,55,57,58,59,60], and low back pain (*p* = 0.7819) [33,35,36,38,40,41,42,44,45,48,49,50,58,59,60]. These results suggest that there is no evidence of publication bias in the studies reviewed. However, when evaluating studies that reported chills (*p* = 0.0294) [37,40,44,46,50,51,52,55,56,57,58,59,60] and arthralgia (*p* = 0.0242) [33,34,35,36,37,38,39,40,41,42,43,44,45,46,48,49,50,51,52,55,56,57,58,59,60], a possible publication bias was found (Table 2).

### 3.4. Clinical Manifestations of Peruvian Patients with Dengue Fever

**Figure 3:**
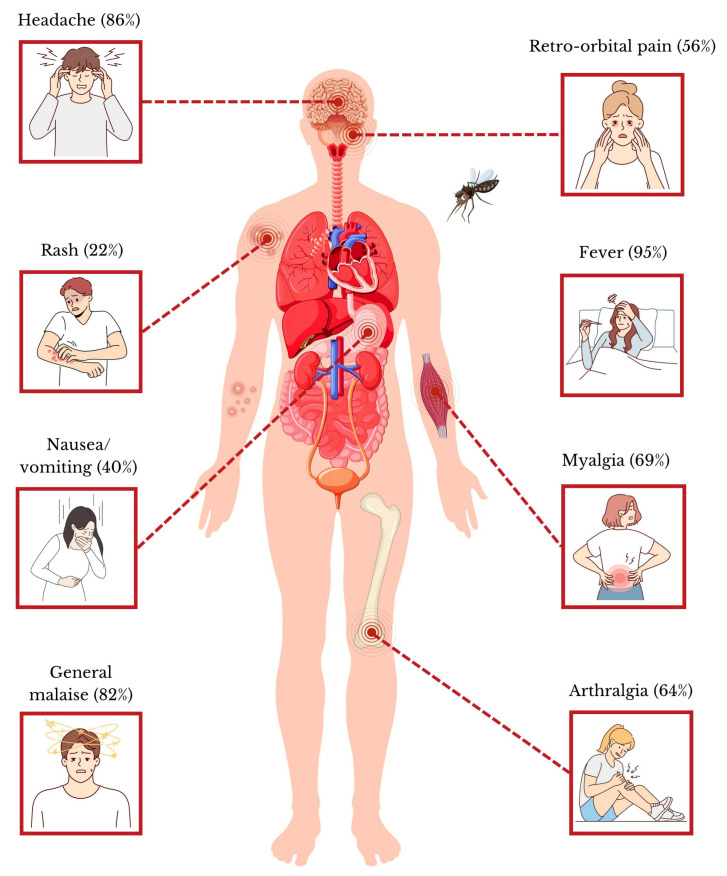
Most frequent clinical manifestations of Peruvian patients with dengue [33,34,35,36,37,38,39,40,41,42,43,44,45,46,47,48,49,50,51,52,53,54,55,56,57,58,59,60].

An analysis of the prevalence of clinical manifestations was performed according to the data in Table S6. The most frequent clinical findings in Peruvian patients with dengue fever were fever at 95% (95% CI: 90–98%; I^2^ = 96%) [34,35,36,37,38,40,41,42,43,44,45,46,47,48,49,50,51,52,54,55,56,57,58,59,60], followed by headache at 86% (95% CI: 80–91%; I^2^ = 96%) [33,34,35,36,37,38,39,40,41,42,43,44,45,46,47,48,49,50,51,52,54,55,56,57,58,59,60], malaise at 82% (95% CI: 71–91%; I^2^ = 93%) [34,36,46,47,51,56,57,58,59,60], myalgia at 69% (95% CI: 58–79%; I^2^ = 97%) [33,34,35,36,38,39,40,41,42,43,44,45,46,47,48,49,50,51,52,54,55,56,58,59,60], and arthralgia at 64% (95% CI: 56–73%; I^2^ = 97%) [33,34,35,36,37,38,39,40,41,42,43,44,45,46,48,49,50,51,52,55,56,57,58,59,60]. The complete pooled prevalence for all other assessed clinical manifestations, including less common symptoms such as melena and jaundice, is detailed in Table 3. In addition, Figure 3 shows the most frequent clinical findings, which support the statements of the World Health Organization [33,34,35,36,37,38,39,40,41,42,43,44,45,46,47,48,49,50,51,52,53,54,55,56,57,58,59,60].

## 4. Discussion

In this systematic review and meta-analysis, we compiled updated data on the prevalence of clinical manifestations of dengue in the Peruvian population. The results of our study allow for more effective follow-up of dengue patients, facilitate early detection of complications, and improve clinical outcomes. In addition, knowing the common manifestations of dengue helps to make prevention campaigns more targeted, improving early detection and medical care. These data are crucial for resource planning and control measures in Peru and can guide health policy makers in strengthening surveillance and response systems. The prevalence of specific symptoms guides the allocation of resources for diagnosis and treatment, allowing the development of more effective response protocols and better training of health professionals [21]. We will then critically examine these findings and discuss their relevance for public health and clinical practice.

Our results indicate that the most prevalent clinical manifestations were fever (95%), headache (86%), malaise (82%), myalgia (69%), arthralgia (64%), and retro-orbital pain (56%) (Figure 3). A meta-analysis conducted in the Pacific Islands by Kharwadkar et al. reported similar results, highlighting that the most frequent symptoms referred for dengue cases were fever (97.45%), headache (81.62%), myalgia (74.20%), chills (65.29%), and arthralgia (57.47%) [62]. However, a meta-analysis by Asish PR et al. reported that about 59.26% of dengue cases are asymptomatic and may play an important role in disease transmission [63]. In addition, dengue viruses cause a nonspecific acute febrile illness in about 25% of those infected, and about 5% of those with symptoms develop severe dengue, characterized by plasma leakage, hemorrhage, shock, or severe organ damage. Infants, the elderly, pregnant women, people with a second dengue infection, and those with certain underlying conditions are at increased risk of experiencing severe dengue [64].

The variety of clinical manifestations observed can provide guidance on the severity levels of dengue, thus facilitating early diagnosis and appropriate treatment [21,65]. According to PAHO, there are three levels of severity, each with characteristic symptoms [66]. Dengue without alarm signs includes fever, nausea, vomiting, rash, headache, retroorbital pain, myalgia, arthralgia, petechiae or positive tourniquet test, and leukopenia. Dengue with alarm signs presents with severe abdominal pain, persistent vomiting, fluid accumulation, mucosal bleeding, lethargy or irritability, lipothymia, hepatomegaly >2 cm, and a progressive increase in hematocrit. Finally, severe dengue is characterized by shock or respiratory distress due to severe plasma extravasation, severe bleeding, and severe organ involvement, such as liver damage, central nervous system (altered consciousness), heart (myocarditis), or other organs (Figure S3) [66].

A meta-analysis conducted in Latin America by Paraná VC et al. reported that risk factors associated with severe dengue include secondary dengue infection, female sex, white or Caucasian ethnicity, and certain specific signs and symptoms, such as headache, myalgia and/or arthralgia, vomiting and nausea, abdominal pain or tenderness, diarrhea, prostration, lethargy, and fatigue, among others [2]. Peres IT, et al. described the profile of critically ill dengue patients admitted to intensive care units in Brazil. Significant risk factors for complications included age ≥ 80 years, chronic kidney disease, cirrhosis, low platelet count (<50,000 cells/mm^3^), and high white blood cell count (>7000 cells/mm^3^) [67]. A study conducted by Singh U, et al. identified clinical and biochemical determinants associated with mortality in hospitalized dengue patients. Mortality was significantly associated with advanced age, abdominal pain, difficulty breathing, and weakness. Biochemical predictors included thrombocytopenia, elevated transaminases, bilirubin, and renal markers such as urea and creatinine. Non-survivors also showed a higher prevalence of systemic complications like pleural effusion and ascites [68]. A study by Pham O, et al. examined the clinical phenotypes and outcomes of severe dengue in adults in Vietnam, including 891 cases with a mean age of 29 years. The most prevalent severe phenotype was dengue shock syndrome (DSS), affecting 82.7% of patients. Severe bleeding occurred in 10.1% of cases, and 23.7% experienced organ dysfunction, with liver failure being the most common. Factors associated with recurrent shock episodes included a BMI ≥ 25, illness duration ≤ 5 days, and a history of prior COVID-19 infection [69].

According to the CDC of Peru, as of week 52 of 2025, the departments with the highest incidence of dengue cases are San Martín, Cajamarca, Loreto and Amazonas. As of June 2025, *Aedes aegypti*, the dengue vector, had been reported in 24 regions, 110 provinces, and a total of 609 districts. The most frequent dengue serotype in Peru is dengue type 2 (Genotype II Cosmopolitan). In addition, 31.29% of the cases have been reported in people between 30 and 59 years of age, and 22.95% of the cases in individuals between 18 and 29 years of age. These data reflect the continued expansion of dengue in the country and the need to intensify vector control and epidemiological surveillance measures (Figure S4) [12].

The diagnosis of dengue can be determined from clinical evaluation and laboratory tests, such as molecular tests (PCR), antibody tests (ELISA), and antigen detection tests (NS1) [21,70]. In Peru, differential diagnosis presents a significant challenge due to overlapping clinical features with other endemic vector-borne diseases such as Zika, chikungunya, malaria, and leishmaniasis [21,71]. This situation is exacerbated by the limited availability of laboratory diagnostic facilities [21].

Furthermore, variability in diagnostic methods (including NS1, IgM, IgG, RT-PCR, and clinical definitions) is influenced not only by the stage of infection at which cases are identified but also by the clinical and epidemiological context guiding diagnostic criteria and test indication. These differences may shape the spectrum of reported symptoms, affecting how dengue manifestations are synthesized across studies. Variability in diagnostic methodologies and case definitions accounts for the heterogeneity observed in the pooled analyses. Factors such as study design, sampling strategies, healthcare settings, and the timing of dengue outbreaks influence clinical prevalence estimates. Consequently, while pooled figures provide a global overview of dengue symptomatology in Peru, these findings should be interpreted with caution; they may reflect specific local epidemiological patterns rather than a uniform national clinical profile.

To address diseases transmitted by insect vectors, the WHO has created the “Global Vector Control Response 2017–2030 (GVCR). This plan provides guidelines for countries to strengthen their vector control strategies, with the aim of preventing diseases and managing outbreaks [72]. The initiative involves restructuring current programs, improving technical capacity, optimizing infrastructure, intensifying surveillance, and mobilizing the community [73,74,75].

Peru is a country with a diversity of subtropical and tropical climates, influenced by two determining factors that significantly modify its ecological conditions: the Andes Mountains and the Humboldt and El Niño Ocean currents. A systematic review proposed by Delrieu M. et al. reported that climate change and increased temperatures favor the expansion of mosquito vectors and diseases such as dengue, Zika, and chikungunya, especially in the long term. Most studies show that temperatures above 28 °C enhance the transmission of these viruses, increasing the risk of outbreaks in temperate regions and increasing the burden in tropical regions [76]. Another systematic review conducted in Latin America and the Caribbean by Santos LLM et al. reported that environmental and socioeconomic factors facilitated vector proliferation and adaptation, and host-related factors were reported to aggravate dengue [77]. A study by Lorenz C, et al. analyzed the influence of climate and heatwaves on dengue transmission in São Paulo and Natal, Brazil. Higher minimum temperatures were associated with an increased risk of dengue, while higher maximum temperatures and total precipitation had a negative effect. Heatwaves reduced the risk in São Paulo by 70%, but had no significant impact in Natal, suggesting that the climate-dengue relationship varies by location [78].

This systematic review and meta-analysis provide valuable insights into the clinical manifestations of dengue in the Peruvian population, yet several limitations must be acknowledged. One of the main constraints is the high heterogeneity observed across the included studies, which may stem from variations in diagnostic criteria, study periods, sample sizes (ranging from 24 to 967 participants), and population characteristics, including age distribution and disease severity. Additionally, differences in dengue serotypes over time could have influenced symptomatology and clinical outcomes, further contributing to the variability of results. The moderate methodological quality of several included studies may undermine the robustness of the pooled estimates. Factors such as small sample sizes, retrospective designs, and incomplete reporting introduce uncertainty and exacerbate inter-study variability. Consequently, although this meta-analysis provides a comprehensive synthesis of the available evidence, the conclusions must be weighed against the potential impact of these methodological limitations and heterogeneity on the reliability of the findings.

The reliance on observational studies introduces potential biases, such as selection bias and unmeasured confounders, which may impact prevalence estimates. Furthermore, the lack of standardized case definitions complicates direct comparisons between studies, while the overrepresentation of data from Piura and Loreto—likely due to higher endemicity, better surveillance infrastructure, or increased research activity in these regions—may limit the generalizability of the findings. Another limitation is the exclusion of gray literature, including conference abstracts, technical reports, and institutional documents. Although this decision was made to prioritize peer-reviewed evidence and ensure methodological consistency, gray literature may contain preliminary or region-specific data that are not yet formally published. Consequently, the exclusion of these sources may have limited the identification of additional studies or recent epidemiological information, potentially affecting the comprehensiveness and timeliness of the evidence synthesis. These methodological limitations highlight the need for further research incorporating broader geographic representation and a longitudinal approach to assess the impact of these findings on clinical practice and public health strategies.

Despite these challenges, this study represents the first systematic review and meta-analysis focused on evaluating dengue clinical manifestations in Peruvian patients. The research rigorously adhered to PRISMA guidelines, employed a specific search strategy for each database, and ensured methodological robustness by conducting all procedures independently by two or more investigators. The findings offer crucial information for updating national management guidelines, particularly in resource-limited settings where early diagnosis and accurate interpretation of diagnostic tests are essential for timely interventions and improved clinical outcomes. Additionally, this research, conducted by a multidisciplinary team, has demonstrated in previous work that it is essential to integrate both quantitative and qualitative synthesis of prevalence studies across different contexts and diseases [79,80,81].

Given the variability in reported symptoms, it is imperative to enhance health personnel training in recognizing clinical signs and interpreting diagnostic results, particularly in areas with limited access to advanced technology. Strengthening these capacities will enable more effective management strategies adapted to local realities, ultimately contributing to a better response to future dengue outbreaks. Moreover, further research—such as meta-regression and sensitivity analyses—could help clarify whether pooled estimates accurately represent the clinical profile of dengue or if methodological factors have influenced the observed trends.

## 5. Conclusions

Given that dengue is endemic in our country and considering the various outbreaks that have occurred in recent years, our data show a high prevalence of variability in the clinical manifestations of the disease. The most common manifestations in Peruvian patients include fever, headache, malaise, myalgia, arthralgia, and retro-orbital pain. However, it should be acknowledged that the heterogeneity in symptom presentation may be due to factors such as age distribution, disease severity, and differences in diagnostic criteria, which limit the generalizability of our results.

This study highlights the need to strengthen epidemiological surveillance, as well as to optimize diagnostic and treatment protocols in endemic areas of Peru. Additionally, it is essential to promote educational campaigns on dengue prevention and management and conduct further research to better understand the factors contributing to symptom heterogeneity. These measures will not only improve the response to outbreaks but also address the methodological challenges identified, contributing to a sustained reduction in dengue incidence in the country and fostering broader, standardized data collection at the regional and national levels.

## Figures and Tables

**Table 1:** Summary of dengue studies included in the review.

Authors	Year	Studio Type	Sample	Location	M/F	Age (Years)	Dengue Subtype	Study Period	Sample Characteristics	Method of Diagnosis	Final Outcome	Data Collection Methods	Study Period
Copaja-Corzo C, et al. [33]	2024	Retrospective cohort	152	Ucayali	72/80	7–17 (63); 18–59 (69); ≥60 (20)	NR	2019 to 2023	General population	IgM ELISA, and dengue NS1 antigen test	Dead (13)	Medical records	2019 to 2023
Luque N, et al. [34]	2023	Retrospective cohort	24	Piura	8/16	Mean: 46	NR	2017	General population	IgM ELISA, IgG ELISA, dengue NS1 antigen test	Dead	Medical records	2017
Ramírez-Orrego L, et al. [35]	2023	Retrospective cohort	53	Amazonas	25/28	Median: 37 (23–53.5)	DENV1: 6% (3) DENV2: 94% (50)	December 2021 to February 2022	General population	Dengue NS1 antigen test, RT-PCR	Recovered	Medical records	December 2021 to February 2022
Tarazona-Castro Y, et al. [36]	2022	Cross-sectional	34	Cajamarca	14/20	11 (3); 12–17 (1); 18–39 (17); 40–59 (9); ≥60 (4)	NR	April 2020 to March 2021	General population	IgM ELISA, IgG ELISA, and RT-PCR	Recovered	Medical records	April 2020 to March 2021
Watts DM, et al. [37]	2022	Retrospective cohort	967	Loreto	504/463	1–14 (114); 15–29 (443); 30–44 (240); >44 (169)	NR	1993 to 1999	General population	IgM ELISA, IgG ELISA, and Clinical criteria	Recovered	Medical records	October 1993 to September 1997
Rodríguez-Gómez JH, et al. [38]	2022	Retrospective cohort	102	San Martín	60/42	Mean: 30.2	NR	2011–2016	General population	Notification data (specific method NR)	Recovered	Medical records	2011 to 2016
Montalvo R, et al. [39]	2022	Retrospective cohort	24	Lima	10/14	Mean: 40	NR	January 2018 to December 2020	General population	IgM ELISA, IgG ELISA, dengue NS1 antigen test, RT-PCR	Recovered (21) Dead (3)	Medical records	2018 to 2020
Del Valle-Mendoza J, et al. [40]	2021	Cross-sectional	84	Piura	42/42	0–19 (17); 20–44 (29); 45–59 (14); >60 (24)	NR	March to August 2016	General population	RT-PCR	Recovered	Medical records	March to August 2016
Gutierrez-Portilla WE, et al. [41]	2021	Retrospective cohort	141	La Libertad	90/51	Mean: 35.5	NR	2012–2017	General population	Notification data (specific method NR)	Recovered	Medical records	2012 to 2017
Aguilar-Luis MA, et al. [42]	2021	Retrospective cohort	136	Cajamarca	NR	NS	DENV2: 11.24% (10/89) DENV3: 77.53% (69/89) Non-typeable DENV: 11.24% (10/89)	January 2017 to June 2017	General population	IgM ELISA, IgG ELISA, dengue NS1 antigen test, RT-PCR	Recovered	Medical records	2013 to 2018
Reátegui A, et al. [43]	2021	Retrospective cohort	44	Ica	16/28	Mean: 38.6	NR	2017	General population	Notification data (specific method NR)	Recovered	Medical records	2017
Del Valle-Mendoza J, et al. [44]	2020	Cross-sectional	32	Cajamarca	22/10	<5 (0); 5–11 (0); 12–17 (1); 18–39 (14); 40–59 (12); ≥60 (5)	DENV2: 6.2% (2) DENV3: 12.5% (4) Non-typeable DENV: 81.3% (26)	February to June 2016	General population	RT-PCR	Recovered	Medical records	February to June 2016
Ruiz Chang WB, et al. [45]	2020	Retrospective cohort	120	La Libertad	NR	Range: 6 to 70	NR	2019	General population	Dengue NS1 antigen test	Recovered	Medical records	January to December 2019
Elson WH, et al. [46]	2020	Retrospective cohort	79	Loreto	38/41	Median: 17 (12–27.5)	DENV2: 96% (76) DENV3: 4% (3)	2016–2019	General population	RT-PCR	Recovered	Medical records	2016 to 2017
Schaber KL, et al. [47]	2019	Retrospective cohort	62	Loreto	35/27	Mean: 17	NR	2019	General population	RT-PCR or viral nucleic acid test positive	Recovered	Interview	2019
Palomares-Reyes C, et al. [48]	2019	Retrospective cohort	69	Huánuco	27/42	0–4 (2), 5–11 (6), 12–17 (9), 18–39 (38), 40–59 (12), and ≥60 (2)	DENV1, DENV2, DENV3, DENV4	December 2015 to March 2016	General population	RT-PCR	Recovered	Medical records	December 2015 to March 2016
Perales Carrasco JCT, et al. [49]	2019	Retrospective cohort	874	Lambayeque	412/462	Mean: 27.6	NR	December 2016 to May 2017	General population	IgM ELISA, IgG ELISA, dengue NS1 antigen test, RT-PCR	Recovered (685) Observation (176) Dead (13)	Medical records	December 2016 to May 2017
Sánchez-Carbonel J, et al. [50]	2018	Cross-sectional	170	Piura	89/81	0–4 (10), 5–19 (40), 20–44 (55), 45–59 (30), and ≥60 (35)	DENV1: 0% (0) DENV2: 57.1% (97) DENV3: 5.3% (9) DENV4: 0% (0) Undetermined: 37.6% (64)	May to August 2016	General population	RT-PCR	Recovered	Medical records	May to August 2016
Halsey ES, et al. [51]	2016	Retrospective cohort	51	Loreto and Madre de Dios	36/15	Mean: 27.8	DENV1: 12% (6) DENV3: 88% (45)	January 2002 to March 2011	General population	RT-PCR	Recovered	Medical records	January 2002 to March 2011
Olkowski S, et al. [52]	2013	Retrospective cohort	194	Loreto	NS	NS	DENV3: 25.7% (50) DENV4: 74.3% (144)	September 2006 to February 2011	General population	RT-PCR	Recovered	Medical records	September 2006 to February 2011
Suárez-Ognio L, et al. [53]	2011	Case and control	175	Loreto	NS	NS	DENV1, DENV2, DENV4	October 2010 to February 2011	General population	IgM ELISA, IgG ELISA, dengue NS1 antigen test, RT-PCR	Recovered	Medical records	October 2010 to February 2011
Fiestas Solórzano V, et al. [54]	2011	Retrospective cohort	41	Loreto	NR	Mean: 28.4	DENV2: 41% (17/41) DENV4: 5% (2/41) Undetermined: 53.7% (22/41)	2011	General population	IgM ELISA, IgG ELISA, dengue NS1 antigen test, RT-PCR	Observation (38) Dead (3)	Medical records	January to February 2011
Mamani E, et al. [55]	2010	Retrospective cohort	73	Piura	28/45	0–10 (4), 10–20 (15), 20–30 (17), 30–40 (17), 40–50 (11), 50–60 (6), and ≥60 (3)	DENV1: 39.7% (29) DENV3: 46.6% (34) DENV4: 5.5% (4) DENV1 and 3: 8.2% (6)	2008	General population	Dengue NS1 antigen test, RT-PCR	Recovered	Medical records	May to June 2008
Troyes R L, et al. [56]	2006	Retrospective cohort	141	Cajamarca	NR	NR	NR	May 2004 to April 2005	General population	IgM ELISA, dengue NS1 antigen test	Recovered	Medical records	May 2004 to April 2005
Leiva Herrada CH, et al. [57]	2004	Cross-sectional retrospective	31	Piura	NR	<15	NR	August 2000 to April 2001	Pediatric population	IgM ELISA and Clinical criteria	Recovered	Medical records	2004
Mostorino ER, et al. [58]	2002	Retrospective cohort	236	Lima	113/123	0–5 (3), 5–14 (29), 15–19 (27), 20–29 (65), 30–39 (44), 40–49 (28),50–59 (21), and ≥60 (14)	DENV1: 55.1% (130) DENV2: 20.3% (48) DENV3: 23.7% (56) DENV4: 0.8% (2)	2001	General population	IgM ELISA, IgG ELISA, and indirect immunofluorescence test (IFA)	Recovered	Medical records	2001
Moscol E, et al. [59]	2002	Retrospective cohort	92	Piura	37/55	Range: 0–80	NR	January to June 2001	General population	Notification data (specific method NR)	Recovered	Medical records	2001
Phillips I, et al. [60]	1993	Retrospective cohort	217	Loreto	116/101	0–10 (10), 11–20 (31), 21–30 (59), 31–40 (75), 41–50 (21), 51–60 (5), and ≥60 (4)	DENV1 and DENV4	March to July 1990	General population	Notification data (specific method NR)	Recovered	Medical records	1993

NR: Not Reported; NS: Not Specified; ELISA: enzyme-linked immunosorbent assay; RT-PCR: reverse transcriptase polymerase chain reaction; DENV1: dengue type 1; DENV2: dengue type 2; DENV3: dengue type 3; DENV4: dengue type 4.

**Table 2:** Publication bias of the included studies according to the clinical manifestations of Peruvian patients with dengue.

Clinical Manifestations	Studies	Egger’s Test	Publication Bias No or Possible	Supplementary Material
Chills	13	t = −2.50, df = 11, *p*-value = 0.0294	Possible	Figure S2a
Fever	25	t = 0.19, df = 23, *p*-value = 0.8505	No	Figure S2b
Headache	27	t = −1.00, df = 25, *p*-value = 0.3265	No	Figure S2c
Retro-orbital pain	25	t = −1.72, df = 23, *p*-value = 0.0980	No	Figure S2d
Myalgia	25	t = 0.80, df = 23, *p*-value = 0.4298	No	Figure S2e
Arthralgia	25	t = −2.41, df = 23, *p*-value = 0.0242	Possible	Figure S2f
General malaise	10	t = 0.50, df = 8, *p*-value = 0.6333	No	Figure S2g
Nausea/vomiting	25	t = −2.04, df = 23, *p*-value = 0.0530	No	Figure S2h
Abdominal pain	21	t = 1.74, df = 19, *p*-value = 0.0977	No	Figure S2i
Diarrhea	15	t = −0.87, df = 13, *p*-value = 0.3986	No	Figure S2j
Hematemesis	9	⁂	⁂	⁂
Rash	22	t = −0.56, df = 20, *p*-value = 0.5834	No	Figure S2k
Jaundice	7	⁂	⁂	⁂
Low back pain	15	t = −0.28, df = 13, *p*-value = 0.7819	No	Figure S2l
Cough	9	⁂	⁂	⁂
Sore throat	5	⁂	⁂	⁂
Melena	5	⁂	⁂	⁂
Petechiae	9	⁂	⁂	⁂
Ecchymosis	6	⁂	⁂	⁂

⁂ It was not performed because there were fewer than 10 studies.

**Table 3:** Pooled prevalence of clinical manifestations in Peruvian patients diagnosed with dengue.

Clinical Manifestations	Studies	Cases	Sample Size	I^2^ (%)	*p*-Value	Prevalence % (95% CI)	Supplementary Material
Chills	13	1640	2367	99	*p* < 0.01	46 (22–71)	Figure S1a
Fever	25	3724	4067	96	*p* < 0.01	95 (90–98)	Figure S1b
Headache	27	3658	4243	96	*p* < 0.01	86 (80–91)	Figure S1c
Retro-orbital pain	25	2596	4140	97	*p* < 0.01	56 (47–66)	Figure S1d
Myalgia	25	2124	3245	97	*p* < 0.01	69 (58–79)	Figure S1e
Arthralgia	25	2991	4140	97	*p* < 0.01	64 (56–73)	Figure S1f
General malaise	10	760	967	93	*p* < 0.01	82 (71–91)	Figure S1g
Nausea/vomiting	25	1557	3341	94	*p* < 0.01	40 (33–48)	Figure S1h
Abdominal pain	21	845	2921	98	*p* < 0.01	33 (21–45)	Figure S1i
Diarrhea	15	444	2317	90	*p* < 0.01	16 (11–22)	Figure S1j
Hematemesis	9	65	1474	83	*p* < 0.01	06 (02–10)	Figure S1k
Rash	22	936	3797	94	*p* < 0.01	22 (16–28)	Figure S1l
Jaundice	7	35	802	78	*p* < 0.01	03 (01–07)	Figure S1m
Low back pain	15	1040	2512	98	*p* < 0.01	39 (26–52)	Figure S1n
Cough	9	434	1838	97	*p* < 0.01	10 (03–21)	Figure S1o
Sore throat	5	366	1669	88	*p* < 0.01	23 (16–30)	Figure S1p
Melena	5	11	466	83	*p* < 0.01	01 (00–06)	Figure S1q
Petechiae	9	71	993	82	*p* < 0.01	06 (02–10)	Figure S1r
Ecchymosis	6	21	685	91	*p* < 0.01	03 (00–10)	Figure S1s

Note: Confidence interval (CI).

## Data Availability

All data generated or analyzed during this study are included in this published article.

## Supplementary Materials

The following supporting information can be downloaded at: https://www.mdpi.com/article/10.3390/v18070732/s1, Table S1: PRISMA Checklist (PRISMA 2020 Main Checklist and PRISMA Abstract Checklist); Table S2: The adjusted search terms as per the electronic databases searched; Table S3: Quality of the dengue studies included in the review; Table S4: Database; Table S5: R version 4.2.3. script; Table S6: Summary of studies on clinical manifestations in patients with dengue included in the review; Figure S1: Forest diagram illustrating the combined prevalence of clinical manifestations in Peruvian patients with dengue; Figure S2: Funnel plot and Egger’s test illustrate the publication bias of the included studies; Figure S3: Classification of the severity of dengue according to the Pan American Health Organization; Figure S4: Dengue cases in Peru during 2014–2025 (week 52) were reported by the National Center for Epidemiology, Prevention, and Disease Control of Peru.

## References

[1] Guzman M.G., Halstead S.B., Artsob H., Buchy P., Farrar J., Gubler D.J., Hunsperger E., Kroeger A., Margolis H.S., Martínez E. (2010). Dengue: A Continuing Global Threat. Nat. Rev. Microbiol..

[2] Paraná V.C., Feitosa C.A., da Silva G.C.S., Gois L.L., Santos L.A. (2024). Risk Factors Associated with Severe Dengue in Latin America: A Systematic Review and Meta-Analysis. Trop. Med. Int. Health.

[3] Guzman M.G., Harris E. (2015). Dengue. Lancet.

[4] Paz-Bailey G., Adams L.E., Deen J., Anderson K.B., Katzelnick L.C. (2024). Dengue. Lancet.

[5] León-Figueroa D.A., Abanto-Urbano S., Olarte-Durand M., Nuñez-Lupaca J.N., Barboza J.J., Bonilla-Aldana D.K., Yrene-Cubas R.A., Rodriguez-Morales A.J. (2022). COVID-19 and Dengue Coinfection in Latin America: A Systematic Review. New Microbes New Infect..

[6] Menegon M., Severini F., Toma L., Martignoni M., Di Luca M. (2025). Rapid Molecular Method for Early Detection of the Invasive Mosquito Aedes Aegypti (Linnaeus, 1762) at Points of Entry. Acta Trop..

[7] Dengue. https://www.who.int/news-room/fact-sheets/detail/dengue-and-severe-dengue.

[8] CDC Dengue Symptoms and Treatment|CDC. https://www.cdc.gov/dengue/signs-symptoms/?CDC_AAref_Val=https://www.cdc.gov/dengue/symptoms/index.html.

[9] Dengue: Data and Analysis—PAHO/WHO|Pan American Health Organization. https://www.paho.org/en/arbo-portal/dengue-data-and-analysis.

[10] Dengue: Analysis by Country—PAHO/WHO|Pan American Health Organization. https://www.paho.org/en/arbo-portal/dengue-data-and-analysis/dengue-analysis-country.

[11] Negreiros G.C.L., Vásquez S.R., Hipólito J.V.V. (2024). Características clínicas y situación epidemiológica del dengue en Perú: Una Revisión Sistemática. Rev. del Cuerpo Médico Hosp. Nac. Almanzor Aguinaga Asenjo.

[12] Vigilancia epidemiológica Centro Nacional de Epidemiología Prevención y Control de Enfermedades. https://www.dge.gob.pe/portalnuevo/vigilancia-epidemiologica/.

[13] Arrasco J., Mateo S.Y., Valderrama Y. (2024). Población de áreas de transmisión de dengue y factores demográficos y socioeconómicos. Perú, 2010–2023. Rev. del Cuerpo Médico Hosp. Nac. Almanzor Aguinaga Asenjo.

[14] Llanos-Cuentas A., Altamirano-Quiroz A. (2023). El clima y la epidemia del dengue. Rev. Médica Hered..

[15] Carrillo-Larco R.M., Guzman-Vilca W.C., Leon-Velarde F., Bernabe-Ortiz A., Jimenez M.M., Penny M.E., Gianella C., Leguía M., Tsukayama P., Hartinger S.M. (2022). Peru—Progress in Health and Sciences in 200 Years of Independence. Lancet Reg. Health—Am..

[16] Harris E. (2024). Dengue Cases Surge in Latin America and Caribbean. JAMA.

[17] Hauner A., Aroni-Sierra J., Merino X., Villa C., Torres F., Lagatie O., Talledo M., Ariën K.K., Falconi-Agapito F. (2026). Assessing the Diagnostic Performance of Clinical, Serological and Molecular Approaches to Improve Dengue Case Detection in the Peruvian Amazon. PLoS Negl. Trop. Dis..

[18] de Almeida M.T., Merighi D.G.S., Visnardi A.B., Boneto Gonçalves C.A., Amorim V.M.d.F., Ferrari A.S.dA., de Souza A.S., Guzzo C.R. (2025). Latin America’s Dengue Outbreak Poses a Global Health Threat. Viruses.

[19] Gonzales W.E.G. (2014). Dengue un problema reemergente de Salud Pública. Rev. Científica Ágora.

[20] Page M.J., McKenzie J.E., Bossuyt P.M., Boutron I., Hoffmann T.C., Mulrow C.D., Shamseer L., Tetzlaff J.M., Akl E.A., Brennan S.E. (2021). The PRISMA 2020 Statement: An Updated Guideline for Reporting Systematic Reviews. BMJ.

[21] León-Figueroa D.A., Garcia-Vasquez E.A., Diaz-Torres M., Aguirre-Milachay E., Villanueva-De La Cruz J.P., Saldaña-Cumpa H.M., Valladares-Garrido M.J. (2025). Prevalence of Dengue in Febrile Patients in Peru: A Systematic Review and Meta-Analysis. PLoS ONE.

[22] León-Figueroa D.A., Aguirre-Milachay E., Diaz-Torres M., Failoc-Rojas V.E., Camacho-Neciosup R., Chávarry Isla A.E., Valladares-Garrido M.J. (2025). Epidemiological and Clinical Characteristics of Peruvian Patients with Mpox: A Systematic Review and Meta-Analysis. PLoS ONE.

[23] León-Figueroa D.A., Aguirre-Milachay E., Diaz-Torres M., Villanueva-De La Cruz J.P., Garcia-Vasquez E.A., Failoc-Rojas V.E., Valladares-Garrido M.J. (2025). Oropouche Infection in Peruvian Patients: A Systematic Review and Meta-Analysis. PLoS ONE.

[24] JBI Manual for Evidence Synthesis—JBI Global Wiki. https://jbi-global-wiki.refined.site/space/MANUAL.

[25] Chapter 3: Defining the Criteria for Including Studies and How They Will Be Grouped for the Synthesis. https://training.cochrane.org/handbook/current/chapter-03.

[26] Munn Z., Moola S., Lisy K., Riitano D., Tufanaru C. (2015). Methodological Guidance for Systematic Reviews of Observational Epidemiological Studies Reporting Prevalence and Cumulative Incidence Data. Int. J. Evid. Based Healthc..

[27] Dekkers O.M., Vandenbroucke J.P., Cevallos M., Renehan A.G., Altman D.G., Egger M. (2019). COSMOS-E: Guidance on Conducting Systematic Reviews and Meta-Analyses of Observational Studies of Etiology. PLoS Med..

[28] Información Dengue Instituto de Evaluación de Tecnologías en Salud e Investigación (IETSI). https://ietsi.essalud.gob.pe/info-dengue/.

[29] World Health Organization (2009). Dengue: Guidelines for Diagnosis, Treatment, Prevention and Control: New Edition.

[30] Migliavaca C.B., Stein C., Colpani V., Barker T.H., Ziegelmann P.K., Munn Z., Falavigna M. (2022). Prevalence Estimates Reviews-Systematic Review Methodology Group (PERSyst). Meta-Analysis of Prevalence: I2 Statistic and How to Deal with Heterogeneity. Res. Synth. Methods.

[31] Ioannidis J.P.A., Patsopoulos N.A., Evangelou E. (2007). Uncertainty in Heterogeneity Estimates in Meta-Analyses. BMJ.

[32] Lin L., Chu H. (2018). Quantifying Publication Bias in Meta-Analysis. Biometrics.

[33] Copaja-Corzo C., Flores-Cohaila J., Tapia-Sequeiros G., Vilchez-Cornejo J., Hueda-Zavaleta M., Vilcarromero S., Santana-Téllez T., Parodi J.F., Gomez-Colque S., Benites-Zapata V.A. (2024). Risk Factors Associated with Dengue Complications and Death: A Cohort Study in Peru. PLoS ONE.

[34] Luque N., Cilloniz C., Pons M.J., Donaires F., Albornoz R., Mendocilla-Risco M., Espinoza M. (2023). Características clínicas y epidemiológicas de las muertes por dengue durante un brote en el norte del Perú. Rev. Peru. Med. Exp. Salud Pública.

[35] Ramírez-Orrego L., Rojas L.M., Campos C.J., Gutierrez C., Chenet S.M., Gonzales L., Ramírez-Orrego L., Rojas L.M., Campos C.J., Gutierrez C. (2023). Primer Reporte de Un Brote de Dengue En Balsas, Amazonas, Perú, Durante 2021 y 2022. Rev. Fac. Med. Humana.

[36] Tarazona-Castro Y., Troyes-Rivera L., Martins-Luna J., Cabellos-Altamirano F., Aguilar-Luis M.A., Carrillo-Ng H., Del Valle L.J., Kym S., Miranda-Maravi S., Silva-Caso W. (2022). Detection of SARS-CoV-2 Antibodies in Febrile Patients from an Endemic Region of Dengue and Chikungunya in Peru. PLoS ONE.

[37] Watts D.M., Russell K.L., Wooster M.T., Sharp T.W., Morrison A.C., Kochel T.J., Bautista C.T., Block K., Guevara C., Aguilar P. (2022). Etiologies of Acute Undifferentiated Febrile Illnesses in and near Iquitos from 1993 to 1999 in the Amazon River Basin of Peru. Am. J. Trop. Med. Hyg..

[38] Rodríguez-Gómez J.H. (2022). Dengue con signos de alarma: Características clínicas. Rev. Salud Amaz. Bienestar.

[39] Montalvo R., Diaz-Lazo A., Montalvo M., Ninahuanca C. (2022). Comparación clínica y laboratorial de la fiebre amarilla severa versus dengue grave en Perú. Boletín Malariol. Salud Ambient..

[40] Del Valle-Mendoza J., Palomares-Reyes C., Carrillo-Ng H., Tarazona-Castro Y., Kym S., Aguilar-Luis M.A., Del Valle L.J., Aquino-Ortega R., Martins-Luna J., Peña-Tuesta I. (2021). Leptospirosis in Febrile Patients with Suspected Diagnosis of Dengue Fever. BMC Res. Notes.

[41] Gutierrez-Portilla W.E., Alcalde-Loyola C.C., Aguilar-Urbina E.W. (2021). Características clínicas y epidemiológicas de pacientes adultos con dengue en hospitales de tercer nivel, Perú. Rev. Médica Trujillo.

[42] Aguilar-Luis M.A., Carrillo-Ng H., Kym S., Silva-Caso W., Verne E., Valle L.J.D., Bazán-Mayra J., Zavaleta-Gavidea V., Cornejo-Pacherres D., Tarazona-Castro Y. (2022). Detection of Dengue Virus Serotype 3 in Cajamarca, Peru: Molecular Diagnosis and Clinical Characteristics. Int. J. Infect. Dis..

[43] Reátegui A., Falcón N., Reátegui A., Falcón N. (2021). Características Epidemiológicas y Clínicas de Las Infecciones Por Dengue y Zika Durante El Fenómeno de El Niño Costero de 2017 En Chincha, Perú. Rev. Investig. Vet. Perú.

[44] Del Valle-Mendoza J., Vasquez-Achaya F., Aguilar-Luis M.A., Martins-Luna J., Bazán-Mayra J., Zavaleta-Gavidia V., Silva-Caso W., Carrillo-Ng H., Tarazona-Castro Y., Aquino-Ortega R. (2020). Unidentified Dengue Serotypes in DENV Positive Samples and Detection of Other Pathogens Responsible for an Acute Febrile Illness Outbreak 2016 in Cajamarca, Peru. BMC Res. Notes.

[45] Ruiz Chang W.B. (2020). Caracterización Clínica de Pacientes Con Dengue Provenientes Del Hospital Distrital Santa Isabel—El Porvenir y Del Hospital Distrital Laredo—Laredo, Referidos al Laboratorio de Referencia Regional de La Libertad, Perú—2019. Arnaldoa.

[46] Elson W.H., Reiner R.C., Siles C., Bazan I., Vilcarromero S., Riley-Powell A.R., Kawiecki A.B., Astete H., Hontz R.D., Barker C.M. (2020). Heterogeneity of Dengue Illness in Community-Based Prospective Study, Iquitos, Peru. Emerg. Infect. Dis..

[47] Schaber K.L., Paz-Soldan V.A., Morrison A.C., Elson W.H.D., Rothman A.L., Mores C.N., Astete-Vega H., Scott T.W., Waller L.A., Kitron U. (2019). Dengue Illness Impacts Daily Human Mobility Patterns in Iquitos, Peru. PLoS Negl. Trop. Dis..

[48] Palomares-Reyes C., Silva-Caso W., Del Valle L.J., Aguilar-Luis M.A., Weilg C., Martins-Luna J., Viñas-Ospino A., Stimmler L., Mallqui Espinoza N., Aquino Ortega R. (2019). Dengue Diagnosis in an Endemic Area of Peru: Clinical Characteristics and Positive Frequencies by RT-PCR and Serology for NS1, IgM, and IgG. Int. J. Infect. Dis..

[49] Tito Perales Carrasco J.C., Popuche Cabrera P.L., Cabrejos Sampen G., Díaz-Vélez C., Tito Perales Carrasco J.C., Popuche Cabrera P.L., Cabrejos Sampen G., Díaz-Vélez C. (2019). Perfil Clínico, Epidemiológico y Geográfico de Casos de Dengue Durante El Fenómeno El Niño Costero 2017, Lambayeque-Perú. Rev. Habanera Cienc. Médicas.

[50] Sánchez-Carbonel J., Tantaléan-Yépez D., Aguilar-Luis M.A., Silva-Caso W., Weilg P., Vásquez-Achaya F., Costa L., Martins-Luna J., Sandoval I., Del Valle-Mendoza J. (2018). Identification of Infection by Chikungunya, Zika, and Dengue in an Area of the Peruvian Coast. Molecular Diagnosis and Clinical Characteristics. BMC Res. Notes.

[51] Halsey E.S., Baldeviano G.C., Edgel K.A., Vilcarromero S., Sihuincha M., Lescano A.G. (2016). Symptoms and Immune Markers in Plasmodium/Dengue Virus Co-Infection Compared with Mono-Infection with Either in Peru. PLoS Negl. Trop. Dis..

[52] Olkowski S., Forshey B.M., Morrison A.C., Rocha C., Vilcarromero S., Halsey E.S., Kochel T.J., Scott T.W., Stoddard S.T. (2013). Reduced Risk of Disease during Postsecondary Dengue Virus Infections. J. Infect. Dis..

[53] Suárez-Ognio L., Arrasco J., Casapía M., Sihuincha M., Ávila J., Soto G., Álvarez C., Rodríguez H. (2011). Factores asociados a dengue grave durante la epidemia de dengue en la ciudad de Iquitos, 2010–2011. Rev. Peru. de Epidemiol..

[54] Solórzano V.F., Maldonado M.S., Toscano F.D., Velazco S.D., García M.M., Mamani E., Pretell J.G.d.I.T. (2011). Características clínicas de pacientes internados en el Hospital de Apoyo de Iquitos “César Garayar García” durante la epidemia de dengue, enero-febrero de 2011. Rev. Peru. Med. Exp. Salud Pública.

[55] Mamani E., Figueroa D., García M.P., Garaycochea M.d.C., Pozo E.J. (2010). Infecciones concurrentes por dos serotipos del virus dengue durante un brote en el noroeste de Perú, 2008. Rev. Peru. Med. Exp. Salud Pública.

[56] Rivera L.T., Fuentes L., Troyes M., Canelo L., Garcia M., Anaya E., Tapia R., Zambrano M.C. (2006). Etiología del síndrome febril agudo en la provincia de Jaén, Perú 2004–2005. Rev. Peru. Med. Exp. Salud Pública.

[57] Leiva Herrada C.H., Castro Atarama O., Parra Alejandro J.L. (2004). Aspectos clínicos del síndrome de fiebre del dengue con manifestaciones hemorrágicas en pediatría. Diagnóstico.

[58] Mostorino E.R., Rosas A.Á., Gutiérrez P.V., Anaya R.E., Cobos M., García M.M. (2002). Manifestaciones Clínicas y Distribución Geográfica de Los Serotipos Del Dengue En El Perú—Año 2001. Rev. Peru. Med. Exp. Salud Publica.

[59] Moscol C.E., Moises Alfaro C., García P.J., Suarez J., Sotelo G. (2002). Manifestaciones cutáneas del dengue. Reporte de 92 casos en el Hospitalde Apoyo III Sullana—MINSA durante la epidemia enero a junio del 2001. Dermatol. Peru..

[60] Phillips I., Need J., Escamilla J., Colán E., Sánchez S., Rodríguez M., Vásquez L., Sarmiento J., Betz T., Travassos da Rosa A. (1993). Primer Brote de Dengue Documentado En La Región Amazónica Del Perú. First Doc. Outbreak Dengue Peruv. Amaz. Reg..

[61] Sterrantino A.F. (2024). Observational Studies: Practical Tips for Avoiding Common Statistical Pitfalls. Lancet Reg. Health S. Asia.

[62] Kharwadkar S., Herath N. (2024). Clinical Manifestations of Dengue, Zika and Chikungunya in the Pacific Islands: A Systematic Review and Meta-Analysis. Rev. Med. Virol..

[63] Asish P.R., Dasgupta S., Rachel G., Bagepally B.S., Girish Kumar C.P. (2023). Global Prevalence of Asymptomatic Dengue Infections—A Systematic Review and Meta-Analysis. Int. J. Infect. Dis..

[64] Hernandez-Romieu A.C., Adams L.E., Paz-Bailey G. (2023). Opportunities for Improved Dengue Control in the US Territories. JAMA.

[65] Rodriguez-Morales A.J., León-Figueroa D.A., Sah R., Villamil-Gomez W.E. (2022). Arboviral Diseases and Monkeypox—An Epidemiological Overlapping Differential Diagnosis?. Rev. del Cuerpo Médico Hosp. Nac. Almanzor Aguinaga Asenjo.

[66] Modified Dengue Severity Classification—PAHO/WHO|Pan American Health Organization. https://www.paho.org/en/documents/modified-dengue-severity-classification.

[67] Peres I.T., Ranzani O.T., Bastos L.S.L., Hamacher S., Edinburgh T., Garcia-Gallo E., Bozza F.A. (2025). Clinical Characteristics, Complications and Outcomes of Critically Ill Patients with Dengue in Brazil, 2012-2024: A Nationwide, Multicenter Cohort Study. Int. J. Infect. Dis..

[68] Singh U.P., Mishra D.K., Gangwar P., Kushwaha P. (2025). Clinical Determinants of Mortality in Dengue Fever: A Hospital-Based Study. J. Family Med. Prim. Care.

[69] Pham O.K., Duong T.B., Phan T.V., Truong T.N., Ha D.T.H., Nguyen H.V., Hien V.T.M., Dong T.H.K., Vuong N.L., Yen L.M. (2025). Severe Dengue in Adults: Clinical Features from the 2022 Dengue Outbreak at a Vietnamese Tertiary Hospital. PLoS Negl. Trop. Dis..

[70] Macêdo J.V.L., Júnior A.G.S., Oliveira M.D.L., Andrade C.A.S. (2024). Systematic Review and Meta-Analysis: Assessing the Accuracy of Rapid Immunochromatographic Tests in Dengue Diagnosis. Diagn. Microbiol. Infect. Dis..

[71] Aguilar-Luis M.A., Watson H., Tarazona-Castro Y., Troyes-Rivera L., Cabellos-Altamirano F., Silva-Caso W., Aquino-Ortega R., Carrillo-Ng H., Zavaleta-Gavidia V., Del Valle-Mendoza J. (2023). The Chikungunya Virus: A Reemerging Cause of Acute Febrile Illness in the High Jungle of Northern Peru. PLoS Negl. Trop. Dis..

[72] Global Vector Control Response 2017–2030—PAHO/WHO|Pan American Health Organization. https://www.paho.org/en/documents/global-vector-control-response-2017-2030-0.

[73] Al-Osaimi H.M., Kanan M., Marghlani L., Al-Rowaili B., Albalawi R., Saad A., Alasmari S., Althobaiti K., Alhulaili Z., Alanzi A. (2024). A Systematic Review on Malaria and Dengue Vaccines for the Effective Management of These Mosquito Borne Diseases: Improving Public Health. Hum. Vaccin. Immunother..

[74] Wilson A.L., Courtenay O., Kelly-Hope L.A., Scott T.W., Takken W., Torr S.J., Lindsay S.W. (2020). The Importance of Vector Control for the Control and Elimination of Vector-Borne Diseases. PLoS Negl. Trop. Dis..

[75] Rodriguez-Morales A.J., Puerta-Arias M.C., Husni R., Montenegro-Idrogo J.J., Escalera-Antezana J.P., Alvarado-Arnez L.E., Bonilla-Aldana D.K., Camacho-Moreno G., Mendoza H., Rodriguez-Sabogal I.A. (2025). Infectious Diseases Prevention and Vaccination in Migrants in Latin America: The Challenges of Transit through the Treacherous Darien Gap, Panama. Travel. Med. Infect. Dis..

[76] Delrieu M., Martinet J.-P., O’Connor O., Viennet E., Menkes C., Burtet-Sarramegna V., Frentiu F.D., Dupont-Rouzeyrol M. (2023). Temperature and Transmission of Chikungunya, Dengue, and Zika Viruses: A Systematic Review of Experimental Studies on Aedes Aegypti and Aedes Albopictus. Curr. Res. Parasitol. Vector Borne Dis..

[77] Santos L.L.M., de Aquino E.C., Fernandes S.M., Ternes Y.M.F., Feres V.C.d.R. (2023). Dengue, Chikungunya, and Zika Virus Infections in Latin America and the Caribbean: A Systematic Review. Rev. Panam. Salud Publica.

[78] Lorenz C., Ynoue R.Y., Gioda A., Nogueira T. (2025). Influence of Climate and Heatwaves on Dengue Transmission in Sao Paulo and Natal, Brazil. PLoS ONE.

[79] Gandhi A.P., Gupta P.C., Padhi B.K., Sandeep M., Suvvari T.K., Shamim M.A., Satapathy P., Sah R., León-Figueroa D.A., Rodriguez-Morales A.J. (2023). Ophthalmic Manifestations of the Monkeypox Virus: A Systematic Review and Meta-Analysis. Pathogens.

[80] León-Figueroa D.A., Barboza J.J., Saldaña-Cumpa H.M., Moreno-Ramos E., Bonilla-Aldana D.K., Valladares-Garrido M.J., Sah R., Rodriguez-Morales A.J. (2022). Detection of Monkeypox Virus According to The Collection Site of Samples from Confirmed Cases: A Systematic Review. Trop. Med. Infect. Dis..

[81] León-Figueroa D.A., Barboza J.J., Siddiq A., Sah R., Valladares-Garrido M.J., Adhikari S., Aguirre-Milachay E., Sah S., Rodriguez-Morales A.J. (2024). Prevalence of Computer Vision Syndrome during the COVID-19 Pandemic: A Systematic Review and Meta-Analysis. BMC Public Health.

