# Supplementary material for: Prevalence and Variability of Clinical Manifestations of Dengue in Peru: A Systematic Review and Meta-Analysis of Observational Studies
Source: Viruses. 2026 Jul 2;18(7):732. doi: 10.3390/v18070732 (PMC13431482; doi:10.3390/v18070732)
Supplement: Supplementary file 1 [file viruses-18-00732-s001.zip › viruses-4190798-supplementary.pdf]

Supplementary material

- → **S1 Table.** PRISMA Checklist (PRISMA 2020 Main Checklist and PRIMSA Abstract Checklist).
- → **S2 Table.** The adjusted search terms as per the electronic databases searched.
- → **S3 Table.** Quality of the dengue studies included in the review.
- → **S4 Table.** Database
- → **S5 Table.** R version 4.2.3. script
- → **S6 Table.** Summary of studies on clinical manifestations in patients with dengue included in the review.
- → **S1 Figure.** Forest diagram illustrating the combined prevalence of clinical manifestations in Peruvian patients with dengue.
- → **S2 Figure.** Funnel plot and Egger's test illustrate the publication bias of the included studies.
- → **S3 Figure**. Classification of the severity of dengue according to the Pan American Health Organization.
- → **S4 Figure**. Dengue cases in Peru during 2014–2025 (week 52) were reported by the National Center for Epidemiology, Prevention, and Disease Control of Peru.

S1 Table. PRISMA Checklist (PRISMA 2020 Main Checklist and PRIMSA Abstract Checklist)

PRISMA 2020 Main Checklist

Topic	No.	Item	Location where item is reported
**TITLE**			
**Title**	1	Identify the report as a systematic review.	1
**ABSTRACT**			
**Abstract**	2	See the PRISMA 2020 for Abstracts checklist	
**INTRODUCTION**			
**Rationale**	3	Describe the rationale for the review in the context of existing knowledge.	4
**Objectives**	4	Provide an explicit statement of the objective(s) or question(s) the review addresses.	5
**METHODS**			
**Eligibility criteria**	5	Specify the inclusion and exclusion criteria for the review and how studies were grouped for the syntheses.	5
**Information sources**	6	Specify all databases, registers, websites, organisations, reference lists and other sources searched or consulted to identify studies. Specify the date when each source was last searched or consulted.	5
**Search strategy**	7	Present the full search strategies for all databases, registers and websites, including any filters and limits used.	5-6
**Selection process**	8	Specify the methods used to decide whether a study met the inclusion criteria of the review, including how many reviewers screened each record and each report retrieved, whether they worked independently, and if applicable, details of automation tools used in the process.	6-7
**Data collection process**	9	Specify the methods used to collect data from reports, including how many reviewers collected data from each report, whether they worked independently, any processes for obtaining or confirming data from study investigators, and if applicable, details of automation tools used in the process.	6-7
**Data items**	10a	List and define all outcomes for which data were sought. Specify whether all results that were compatible with each outcome domain in each study were sought (e.g. for all measures, time points, analyses), and if not, the methods used to decide which results to collect.	6-7

Topic	No.	Item	Location where item is reported
	10b	List and define all other variables for which data were sought (e.g. participant and intervention characteristics, funding sources). Describe any assumptions made about any missing or unclear information.	7
**Study risk of bias assessment**	11	Specify the methods used to assess risk of bias in the included studies, including details of the tool(s) used, how many reviewers assessed each study and whether they worked independently, and if applicable, details of automation tools used in the process.	7
**Effect measures**	12	Specify for each outcome the effect measure(s) (e.g. risk ratio, mean difference) used in the synthesis or presentation of results.	7
**Synthesis methods**	13a	Describe the processes used to decide which studies were eligible for each synthesis (e.g. tabulating the study intervention characteristics and comparing against the planned groups for each synthesis (item 5)).	7
	13b	Describe any methods required to prepare the data for presentation or synthesis, such as handling of missing summary statistics, or data conversions.	7
	13c	Describe any methods used to tabulate or visually display results of individual studies and syntheses.	7
	13d	Describe any methods used to synthesize results and provide a rationale for the choice(s). If meta-analysis was performed, describe the model(s), method(s) to identify the presence and extent of statistical heterogeneity, and software package(s) used.	7
	13e	Describe any methods used to explore possible causes of heterogeneity among study results (e.g. subgroup analysis, meta-regression).	7
	13f	Describe any sensitivity analyses conducted to assess robustness of the synthesized results.	-
**Reporting bias assessment**	14	Describe any methods used to assess risk of bias due to missing results in a synthesis (arising from reporting biases).	7
**Certainty assessment**	15	Describe any methods used to assess certainty (or confidence) in the body of evidence for an outcome.	7
**RESULTS**			
**Study selection**	16a	Describe the results of the search and selection process, from the number of records identified in the search to the number of studies included in the review, ideally using a flow diagram.	8
	16b	Cite studies that might appear to meet the inclusion criteria, but which were excluded, and explain why they were excluded.	8

Topic	No.	Item	Location where item is reported
**Study characteristics**	17	Cite each included study and present its characteristics.	8
**Risk of bias in studies**	18	Present assessments of risk of bias for each included study.	9
**Results of individual studies**	19	For all outcomes, present, for each study: (a) summary statistics for each group (where appropriate) and (b) an effect estimate and its precision (e.g. confidence/credible interval), ideally using structured tables or plots.	8-9
**Results of syntheses**	20a	For each synthesis, briefly summarise the characteristics and risk of bias among contributing studies.	9
	20b	Present results of all statistical syntheses conducted. If meta-analysis was done, present for each the summary estimate and its precision (e.g. confidence/credible interval) and measures of statistical heterogeneity. If comparing groups, describe the direction of the effect.	9
	20c	Present results of all investigations of possible causes of heterogeneity among study results.	9
	20d	Present results of all sensitivity analyses conducted to assess the robustness of the synthesized results.	9
**Reporting biases**	21	Present assessments of risk of bias due to missing results (arising from reporting biases) for each synthesis assessed.	9
**Certainty of evidence**	22	Present assessments of certainty (or confidence) in the body of evidence for each outcome assessed.	9
**DISCUSSION**			
**Discussion**	23a	Provide a general interpretation of the results in the context of other evidence.	10-11
	23b	Discuss any limitations of the evidence included in the review.	12
	23c	Discuss any limitations of the review processes used.	12
	23d	Discuss implications of the results for practice, policy, and future research.	12
**OTHER INFORMATION**			
**Registration and protocol**	24a	Provide registration information for the review, including register name and registration number, or state that the review was not registered.	5
	24b	Indicate where the review protocol can be accessed, or state that a protocol was not prepared.	5

Topic	No.	Item	Location where item is reported
	24c	Describe and explain any amendments to information provided at registration or in the protocol.	5
**Support**	25	Describe sources of financial or non-financial support for the review, and the role of the funders or sponsors in the review.	13
**Competing interests**	26	Declare any competing interests of review authors.	13
**Availability of data, code and other materials**	27	Report which of the following are publicly available and where they can be found: template data collection forms; data extracted from included studies; data used for all analyses; analytic code; any other materials used in the review.	13

Note: (–) Not applicable.

PRIMSA Abstract Checklist

Topic	No.	Item	Reported?
**TITLE**			
**Title**	1	Identify the report as a systematic review.	Yes
**BACKGROUND**			
**Objectives**	2	Provide an explicit statement of the main objective(s) or question(s) the review addresses.	Yes
**METHODS**			
**Eligibility criteria**	3	Specify the inclusion and exclusion criteria for the review.	Yes
**Information sources**	4	Specify the information sources (e.g. databases, registers) used to identify studies and the date when each was last searched.	Yes
**Risk of bias**	5	Specify the methods used to assess risk of bias in the included studies.	Yes
**Synthesis of results**	6	Specify the methods used to present and synthesize results.	Yes
**RESULTS**			
**Included studies**	7	Give the total number of included studies and participants and summarise relevant characteristics of studies.	Yes
**Synthesis of results**	8	Present results for main outcomes, preferably indicating the number of included studies and participants for each. If meta-analysis was done, report the summary estimate and confidence/credible interval. If comparing groups, indicate the direction of the effect (i.e. which group is favoured).	Yes
**DISCUSSION**			
**Limitations of evidence**	9	Provide a brief summary of the limitations of the evidence included in the review (e.g. study risk of bias, inconsistency and imprecision).	Yes
**Interpretation**	10	Provide a general interpretation of the results and important implications.	Yes
**OTHER**			
**Funding**	11	Specify the primary source of funding for the review.	Yes
**Registration**	12	Provide the register name and registration number.	Yes

From: Page MJ, McKenzie JE, Bossuyt PM, Boutron I, Hoffmann TC, Mulrow CD, et al. The PRISMA 2020 statement: an updated guideline for reporting systematic reviews. MetaArXiv. 2020, September 14. DOI: 10.31222/osf.io/v7gm2. For more information, visit: www.prisma-statement.org

S2 Table. The adjusted search terms as per the electronic databases searched.

**PubMed**			
	#1	(Dengue[Title/Abstract] OR "Breakbone Fever"[Title/Abstract] OR "Fever, Breakbone"[Title/Abstract] OR "Classical Dengue Fever"[Title/Abstract] OR "Break-Bone Fever"[Title/Abstract] OR "Break Bone Fever"[Title/Abstract] OR "Dengue Fever"[Title/Abstract] OR "Fever, Dengue"[Title/Abstract] OR "Classical Dengue"[Title/Abstract])	30,833
	#2	Peru[Title] OR Peruvian[Title]	9,680
	#3	#1 AND #2	128
**Scopus**			
	#1	TITLE(Dengue OR "Breakbone Fever" OR "Classical Dengue Fever" OR "Break-Bone Fever" OR "Break Bone Fever" OR "Dengue Fever" OR "Classical Dengue")	25,635
	#2	TITLE(Peru OR Peruvian)	34,999
	#3	#1 AND #2	122
**Embase**			
	#1	('dengue'/exp).ti,ab.	42,461
	#2	(' Peru' OR 'Peruvian').ti,ab.	27,021
	#3	#1 AND #2	158
**Web of Science**			
	#1	TI=(Dengue OR "Breakbone Fever" OR "Fever, Breakbone" OR "Classical Dengue Fever" OR "Break-Bone Fever" OR "Break Bone Fever" OR "Dengue Fever" OR "Fever, Dengue" OR "Classical Dengue")	21,108
	#2	TI=(Peru OR Peruvian)	24,824
	#3	#1 AND #2	120
**ScienceDirect**			
	#1	Title, abstract, keywords: (Dengue)	12,150
	#2	Title, abstract, keywords: (Peru OR Peruvian)	11,637
	#3	#1 AND #2	56
**Google Scholar**			
	#1	allintitle: "Dengue"	146,000

#2	allintitle: "Peru OR Peruvian"	164,000
#3	#1 AND #2	245
**Virtual Health Library (VHL)**		
#1	ti:(Dengue)	26,784
#2	ti:(Peru OR Peruvian)	20,410
#3	#1 AND #2	283
**Scielo**		
#1	(ti:(*dengue))	1,842
#2	(ti:(*Peru))	5, 853
#3	#1 AND #2	35
**TOTAL**	PubMed (n =128); Scopus (n=122); Web of Sciences(n=120); Embase (n=158); ScienceDirect (56); Google Scholar (245); Virtual Health Library (283); and Scielo (35)	1,147

⁂Updated through January 12, 2025.

S3 Table. Quality of the dengue studies included in the review

Authors	Year	Eligibility criteria	Study subjects and the setting	Exposure measured in a valid and reliable way 'gold standard'	A specified diagnosis or definition	Confounding factors	Dealing with confounding factors	Outcomes measured in a valid and reliable way	Appropriate statistical analysis	Scores (8)	Quality (high, moderate, low)
**Copaja-Corzo C,** et al. **(1)**	2024	Yes	Yes	Yes	Yes	Unclear	NA	Yes	Yes	6	Moderate
**Luque N, et al. (2)**	2023	Yes	Yes	Yes	Yes	Unclear	NA	Yes	Yes	6	Moderate
**Ramírez-Orrego L, et al. (3)**	2023	Yes	Yes	Yes	Yes	Unclear	NA	Yes	Yes	6	Moderate
**Tarazona-Castro Y,** et al. **(4)**	2022	Yes	Yes	Yes	Yes	Unclear	NA	Yes	Yes	6	Moderate
**Watts DM,** et al. **(5)**	2022	Yes	Yes	Yes	Yes	Unclear	NA	Yes	Yes	6	Moderate
**Rodríguez-Gómez JH, et al. (6)**	2022	Yes	Yes	Yes	Yes	Unclear	NA	Yes	Yes	6	Moderate
**Montalvo R, et al. (7)**	2022	Yes	Yes	Yes	Yes	Unclear	NA	Yes	Yes	6	Moderate
**Del Valle-Mendoza J,** et al. **(8)**	2021	Yes	Yes	Yes	Yes	Unclear	NA	Yes	Yes	6	Moderate
**Gutierrez-Portilla WE, et al. (9)**	2021	Yes	Yes	Yes	Yes	Unclear	NA	Yes	Yes	6	Moderate
**Aguilar-Luis MA, et al. (10)**	2021	Yes	Yes	Yes	Yes	Unclear	NA	Yes	Yes	6	Moderate

**ReáteguiA, et al. (11)**	2021	Yes	Yes	Yes	Yes	Unclear	NA	Yes	Yes	6	Moderate
**Del Valle-Mendoza J, et al. (12)**	2020	Yes	Yes	Yes	Yes	Unclear	NA	Yes	Yes	6	Moderate
**Ruiz Chang WB, et al. (13)**	2020	Yes	Yes	Yes	Yes	Unclear	NA	Yes	Yes	6	Moderate
**Elson WH,et al. (14)**	2020	Yes	Yes	Yes	Yes	Unclear	NA	Yes	Yes	6	Moderate
**Schaber KL, et al. (15)**	2019	Yes	Yes	Yes	Yes	Unclear	NA	Yes	Yes	6	Moderate
**Palomares-Reyes C, et al. (16)**	2019	Yes	Yes	Yes	Yes	Unclear	NA	Yes	Yes	6	Moderate
**Perales Carrasco JCT, et al. (17)**	2019	Yes	Yes	Yes	Yes	Unclear	NA	Yes	Yes	6	Moderate
**Sánchez-Carbonel J, et al. (18)**	2018	Yes	Yes	Yes	Yes	Unclear	NA	Yes	Yes	6	Moderate
**Halsey ES, et al. (19)**	2016	Yes	Yes	Yes	Yes	Unclear	NA	Yes	Yes	6	Moderate
**Olkowski S,** et al. **(20)**	2013	Yes	Yes	Yes	Yes	Unclear	NA	Yes	Yes	6	Moderate
**Suárez-Ognio L, et al. (21)**	2011	Yes	Yes	Yes	Yes	Unclear	NA	Yes	Yes	6	Moderate
**Fiestas Solórzano V, et al. (22)**	2011	Yes	Yes	Yes	Yes	Unclear	NA	Yes	Yes	6	Moderate
**Mamani E, et al. (23)**	2010	Yes	Yes	Yes	Yes	Unclear	NA	Yes	Yes	6	Moderate

**Troyes R L, et al. (24)**	2006	Yes	Yes	Yes	Yes	Unclear	NA	Yes	Yes	6	Moderate
**Leiva Herrada CH, et al. (25)**	2004	Yes	Yes	Yes	Yes	Unclear	NA	Yes	Yes	6	Moderate
**Mostorino ER, et al. (26)**	2002	Yes	Yes	Yes	Yes	Unclear	NA	Yes	Yes	6	Moderate
**Moscol E, et al. (27)**	2002	Yes	Yes	Yes	Yes	Unclear	NA	Yes	Yes	6	Moderate
**Phillips I, et al. (28)**	1993	Yes	Yes	Yes	Yes	Unclear	NA	Yes	Yes	6	Moderate

NA: Not assessed; **Reference:** Moola S, Munn Z, Tufanaru C, Aromataris E, Sears K, Sfetcu R, Currie M, Qureshi R, Mattis P, Lisy K, Mu P-F Chapter 7: Systematic reviews of etiology and risk: JBI; 2020. Available from: https://synthesismanual.jbi.global

S4 Table. Database

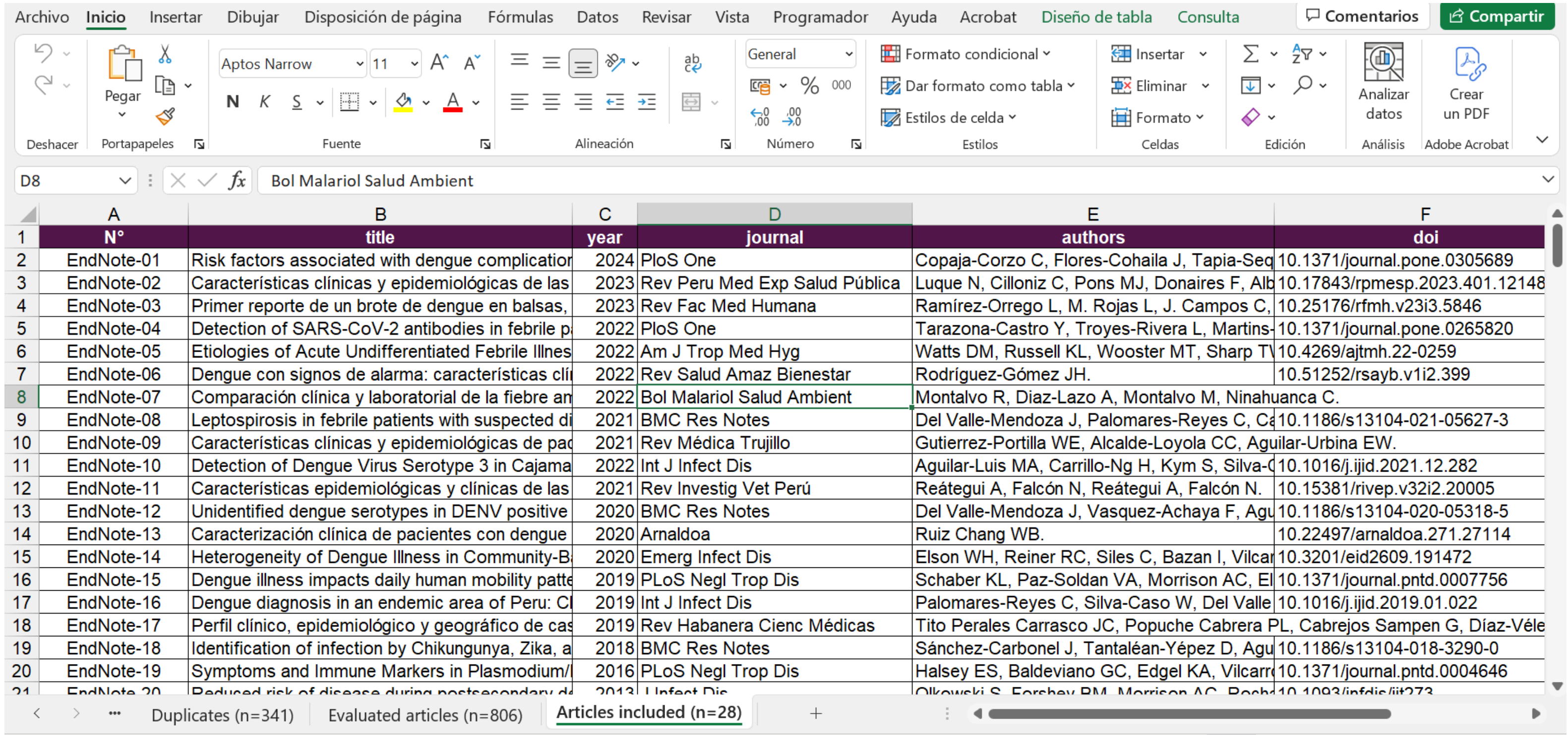

	A	B	C	D	E	F
1	N°	title	year	journal	authors	doi
2	EndNote-01	Risk factors associated with dengue complicatior	2024	PloS One	Copaja-Corzo C, Flores-Cohaila J, Tapia-Seq	10.1371/journal.pone.0305689
3	EndNote-02	Características clínicas y epidemiológicas de las	2023	Rev Peru Med Exp Salud Pública	Luque N, Cilloniz C, Pons MJ, Donaires F, Alb	10.17843/rpmesp.2023.401.12148
4	EndNote-03	Primer reporte de un brote de dengue en balsas,	2023	Rev Fac Med Humana	Ramírez-Orrego L, M. Rojas L, J. Campos C,	10.25176/rfmh.v23i3.5846
5	EndNote-04	Detection of SARS-CoV-2 antibodies in febrile p	2022	PloS One	Tarazona-Castro Y, Troyes-Rivera L, Martins-	10.1371/journal.pone.0265820
6	EndNote-05	Etiologies of Acute Undifferentiated Febrile Illnes	2022	Am J Trop Med Hyg	Watts DM, Russell KL, Wooster MT, Sharp T\	10.4269/ajtmh.22-0259
7	EndNote-06	Dengue con signos de alarma: características clí	2022	Rev Salud Amaz Bienestar	Rodríguez-Gómez JH.	10.51252/rsayb.v1i2.399
8	EndNote-07	Comparación clínica y laboratorial de la fiebre an	2022	Bol Malariol Salud Ambient	Montalvo R, Diaz-Lazo A, Montalvo M, Ninahuanca C.	
9	EndNote-08	Leptospirosis in febrile patients with suspected di	2021	BMC Res Notes	Del Valle-Mendoza J, Palomares-Reyes C, Ca	10.1186/s13104-021-05627-3
10	EndNote-09	Características clínicas y epidemiológicas de pac	2021	Rev Médica Trujillo	Gutierrez-Portilla WE, Alcalde-Loyola CC, Aguilar-Urbina EW.	
11	EndNote-10	Detection of Dengue Virus Serotype 3 in Cajama	2022	Int J Infect Dis	Aguilar-Luis MA, Carrillo-Ng H, Kym S, Silva-C	10.1016/j.ijid.2021.12.282
12	EndNote-11	Características epidemiológicas y clínicas de las	2021	Rev Investig Vet Perú	Reátegui A, Falcón N, Reátegui A, Falcón N.	10.15381/rivep.v32i2.20005
13	EndNote-12	Unidentified dengue serotypes in DENV positive	2020	BMC Res Notes	Del Valle-Mendoza J, Vasquez-Achaya F, Agu	10.1186/s13104-020-05318-5
14	EndNote-13	Caracterización clínica de pacientes con dengue	2020	Arnaldoa	Ruiz Chang WB.	10.22497/arnaldoa.271.27114
15	EndNote-14	Heterogeneity of Dengue Illness in Community-B	2020	Emerg Infect Dis	Elson WH, Reiner RC, Siles C, Bazan I, Vilcar	10.3201/eid2609.191472
16	EndNote-15	Dengue illness impacts daily human mobility patte	2019	PLoS Negl Trop Dis	Schaber KL, Paz-Soldan VA, Morrison AC, El	10.1371/journal.pntd.0007756
17	EndNote-16	Dengue diagnosis in an endemic area of Peru: Cl	2019	Int J Infect Dis	Palomares-Reyes C, Silva-Caso W, Del Valle	10.1016/j.ijid.2019.01.022
18	EndNote-17	Perfil clínico, epidemiológico y geográfico de cas	2019	Rev Habanera Cienc Médicas	Tito Perales Carrasco JC, Popuche Cabrera PL, Cabrejos Sampen G, Díaz-Véle	
19	EndNote-18	Identification of infection by Chikungunya, Zika, a	2018	BMC Res Notes	Sánchez-Carbonel J, Tantaléan-Yépez D, Agu	10.1186/s13104-018-3290-0
20	EndNote-19	Symptoms and Immune Markers in Plasmodium/l	2016	PLoS Negl Trop Dis	Halsey ES, Baldeviano GC, Edgel KA, Vilcarr	10.1371/journal.pntd.0004646
21	EndNote-20	Reduced risk of disease during postsecondary d	2013	J Infect Dis	Olkowski S, Forshey BM, Morrison AC, Rocha	10.1093/infdis/jit273

Duplicates (n=341) Evaluated articles (n=806) Articles included (n=28)

León-Figueroa, Darwin Anthony (2026). S4 Table. Database. figshare. Dataset. https://doi.org/10.6084/m9.figshare.31378942.v1

S5 Table. R version 4.2.3. script

Import database

Environment – From Excel – Import Excel Data - Import

Calculate the prevalence of clinical manifestations.

```
library(meta)
library(readxl)
data_prevalence<- Database name
mtprop=metaprop(event= Type of clinical manifestations,
                n= Sample_ Study,
                studlab=paste(Study,Year),
                data=data_prevalence,
                method.tau = "DL",
                method = "Inverse",
                method.ci = "SACC",
                sm="PFT")
mtprop
forest(mtprop,
          comb.fixed=FALSE,
      common = FALSE)
```

An example is given:

Database name: **Base_Fever**

Study	Year	Sample	Fever
**Luque N, et al.**	2023	24	21
**Ramírez-Orrego L, et al.**	2023	53	47
**Tarazona-Castro Y, et al.**	2022	34	34
**Watts DM, et al.**	2022	967	947
**Rodríguez-Gómez JH, et al.**	2022	102	102
**Del Valle-Mendoza J, et al.**	2021	84	84
**Gutierrez-Portilla WE, et al.**	2021	141	90
**Aguilar-Luis MA, et al.**	2021	136	136
**ReáteguiA, et al.**	2021	44	5
**Del Valle-Mendoza J, et al.**	2020	32	32
**Ruiz Chang WB, et al.**	2020	120	96
**Elson WH,et al.**	2020	79	59
**Schaber KL, et al.**	2019	62	58
**Palomares-Reyes C, et al.**	2019	69	69
**Perales Carrasco JCT, et al.**	2019	874	717
**Sánchez-Carbonel J, et al.**	2018	170	170
**Halsey ES, et al.**	2016	51	51
**Olkowski S, et al.**	2013	194	192
**Fiestas Solórzano V, et al.**	2011	41	37
**Mamani E, et al.**	2010	73	73
**Troyes R L, et al.**	2006	141	141
**Leiva Herrada CH, et al.**	2004	31	31
**Mostorino ER, et al.**	2002	236	236

**Moscol E, et al.**	2002	92	89
**Phillips I, et al.**	1993	217	207

Code in R version 4.2.3

```
library(meta)
library(readxl)
data_prevalence<- Base_Fever
mtprop=metaprop(event= Fever,
                n= Sample,
                studlab=paste(Study,Year),
                data=data_prevalence,
                method.tau = "DL",
                method = "Inverse",
                method.ci = "SACC",
                sm="PFT")
mtprop
forest(mtprop,
       comb.fixed=FALSE,
    common = FALSE)
```

Results

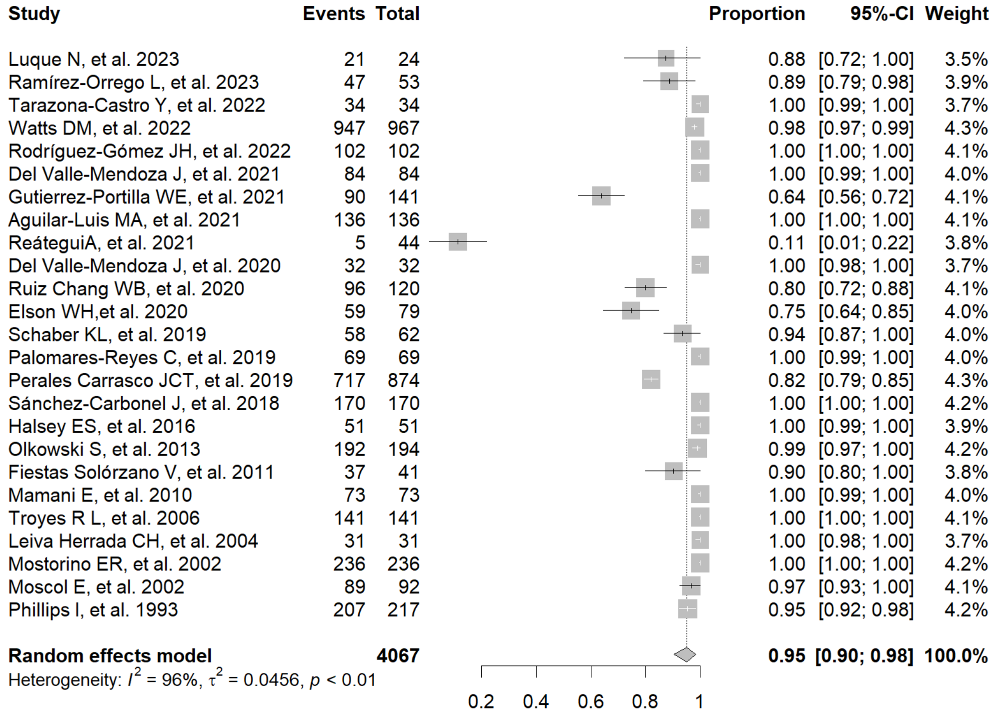

Funnel plot and Egger's test

```
library(meta)
library(readxl)
par(mar = c(5, 5, 2, 2))
data_prevalence<- Database name
mtprop=metaprop(event= Type of clinical manifestations,
                n= Sample_ Study,
                studlab=paste(Study,Year),
```

```
                              data=data_prevalence,
                              method.tau = "DL",
                              method = "Inverse",
                              method.ci = "SACC",
                              sm="PFT")
par(mar = c(5, 5, 2, 2))
funnel(mtprop, comb.fixed = FALSE, common = FALSE)
par(mar = c(5, 5, 2, 2))
egger_test <- metabias(mtprop)
print(egger_test)
```

An example is given:

Database name: **Base_Fever**

Study	Year	Sample	Fever
**Luque N, et al.**	2023	24	21
**Ramírez-Orrego L, et al.**	2023	53	47
**Tarazona-Castro Y, et al.**	2022	34	34
**Watts DM, et al.**	2022	967	947
**Rodríguez-Gómez JH, et al.**	2022	102	102
**Del Valle-Mendoza J, et al.**	2021	84	84
**Gutierrez-Portilla WE, et al.**	2021	141	90
**Aguilar-Luis MA, et al.**	2021	136	136
**ReáteguiA, et al.**	2021	44	5
**Del Valle-Mendoza J, et al.**	2020	32	32
**Ruiz Chang WB, et al.**	2020	120	96
**Elson WH,et al.**	2020	79	59
**Schaber KL, et al.**	2019	62	58
**Palomares-Reyes C, et al.**	2019	69	69
**Perales Carrasco JCT, et al.**	2019	874	717
**Sánchez-Carbonel J, et al.**	2018	170	170
**Halsey ES, et al.**	2016	51	51
**Olkowski S, et al.**	2013	194	192
**Fiestas Solórzano V, et al.**	2011	41	37
**Mamani E, et al.**	2010	73	73
**Troyes R L, et al.**	2006	141	141
**Leiva Herrada CH, et al.**	2004	31	31
**Mostorino ER, et al.**	2002	236	236
**Moscol E, et al.**	2002	92	89
**Phillips I, et al.**	1993	217	207

Code in R version 4.2.3

```
library(meta)
library(readxl)
par(mar = c(5, 5, 2, 2))
data_prevalence<- Base_Fever
mtprop=metaprop(event= Fever,
                n= Sample,
```

```
                              studlab=paste(Study,Year),
                              data=data_prevalence,
                              method.tau = "DL",
                              method = "Inverse",
                              method.ci = "SACC",
                              sm="PFT")
par(mar = c(5, 5, 2, 2))
funnel(mtprop, comb.fixed = FALSE, common = FALSE)
par(mar = c(5, 5, 2, 2))
egger_test <- metabias(mtprop)
print(egger_test)
```

Results

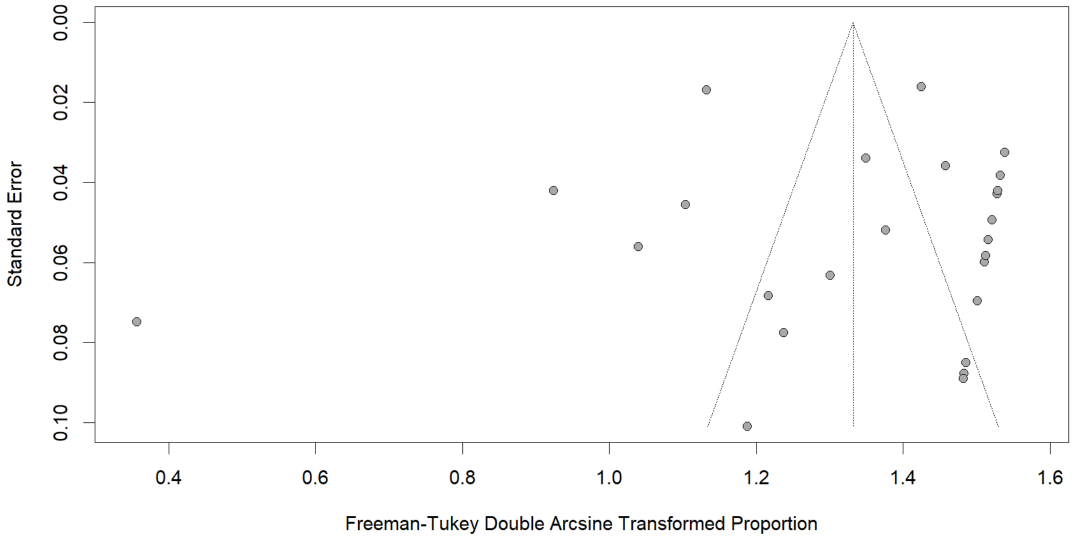

Test result: t = 0.19, df = 23, p-value = 0.8505

S6 Table. Summary of studies on clinical manifestations in patients with dengue included in the review.

Authors	Year	Sample	Chills	Fever	Headache	Retro-orbital pain	Myalgia	Arthralgia	General malaise	Nausea/vomiting	Abdominal pain	Diarrhea	Hematemesis	Rash	Jaundice	Low back pain	Cough	Sore throat	Melena	Petechiae	Ecchymosis
**Copaja-Corzo C, et al.** (1)	2024	152	NS	NS	107	31	106	100	NR	105	113	41	NR	23	NR	23	NR	NR	NR	NR	NR
**Luque N, et al.** (2)	2023	24	NR	21	8	3	1	8	9	14	11	5	1	1	2	NR	NR	NR	NR	2	NR
**Ramírez-Orrego L, et al.** (3)	2023	53	NR	47	48	38	48	40	NR	29	NR	NR	NR	11	NR	35	NR	NR	NR	NR	NR
**Tarazona-Castro Y, et al.** (4)	2022	34	NR	34	30	16	26	9	21	8	NS	NR	NR	7	NR	11	NR	NR	NR	NR	NR
**Watts DM, et al.** (5)	2022	967	880	947	945	818	NS	859	NR	535	NR	213	NR	244	NR	NR	303	185	NR	NR	NR
**Rodríguez-Gómez JH, et al.** (6)	2022	102	NR	102	58	20	68	68	NR	17	73	32	8	13	NR	16	NR	9	9	4	3
**Montalvo R, et al.** (7)	2022	24	NR	NR	14	5	14	5	NR	10	8	6	4	7	NR	NR	NR	NR	NR	NR	13
**Del Valle-Mendoza J, et al.** (8)	2021	84	1	84	73	60	74	71	NR	40	3	NR	NR	16	0	54	1	NR	0	1	0
**Gutierrez-Portilla WE, et al.** (9)	2021	141	NR	90	108	61	100	93	NR	75	83	NR	NR	39	12	20	NR	NR	NR	NR	NR
**Aguilar-Luis MA, et al.** (10)	2021	136	NR	136	120	68	93	89	NR	33	21	13	NR	28	NR	37	NR	NR	NR	NR	NR

**ReáteguiA, et al.** (11)	2021	44	NR	5	43	22	35	23	NR	1	NR	NR	NR	NR	NR	NR	NR	NR	NR	NR	NR
**Del Valle-Mendoza J, et al.** (12)	2020	32	1	32	13	7	7	6	NR	3	1	1	NR	3	NR	3	0	NR	NR	NR	NR
**Ruiz Chang WB, et al.** (13)	2020	120	NR	96	113	102	111	108	NR	67	NR	NR	NR	NR	NR	88	NR	NR	NR	NR	NR
**Elson WH,et al.** (14)	2020	79	44	59	55	37	36	28	77	11	12	7	NR	NR	NR	NR	NR	NR	NR	NR	NR
**Schaber KL, et al.** (15)	2019	62	NR	58	57	NR	53	NR	62	NR	NR	NR	NR	NR	NR	NR	NR	NR	NR	NR	NR
**Palomares-Reyes C, et al.** (16)	2019	69	NR	69	67	48	65	63	NR	36	4	NR	0	18	0	36	NR	NR	0	0	0
**Perales Carrasco JCT, et al.** (17)	2019	874	NR	717	661	485	545	609	NR	NR	72	NR	21	213	NR	391	NR	NR	NR	NR	NR
**Sánchez-Carbonel J, et al.** (18)	2018	170	1	170	152	118	147	143	NR	86	7	NR	NR	26	NR	105	1	50	0	3	1
**Halsey ES, et al.** (19)	2016	51	48	51	51	40	45	48	50	36	15	7	NR	2	NR	NR	5	NR	NR	NR	NR
**Olkowski S, et al.** (20)	2013	194	186	192	189	159	157	160	NR	118	99	59	NR	149	NR	NR	60	60	NR	24	NR
**Suárez-Ognio L, et al.** (21)	2011	175	NR	NR	NR	NR	NR	NR	NR	60	109	5	NR	NR	9	NR	NR	NR	NR	NR	NR
**Fiestas Solórzano V, et al.** (22)	2011	41	NR	37	33	NR	31	NR	NR	25	31	17	3	19	NR	NR	NR	NR	2	5	NR

**Mamani E, et al.** (23)	2010	73	46	73	73	61	69	57	NR	28	33	6	1	15	7	NR	10	NR	NR	10	NR
**Troyes R L, et al.** (24)	2006	141	101	141	101	104	42	37	96	NR	32	NR	NR	NR	NR	NR	NR	NR	NR	NR	NR
**Leiva Herrada CH, et al.** (25)	2004	31	5	31	30	27	NR	25	27	13	18	1	8	7	NR	NR	1	NR	NR	NR	NR
**Mostorino ER, et al.** (26)	2002	236	159	236	225	167	12	158	170	90	78	31	19	41	5	15	53	62	NR	22	4
**Moscol E, et al.** (27)	2002	92	1	89	85	36	66	16	71	7	22	NR	NR	27	NR	36	NR	NR	NR	NR	NR
**Phillips I, et al.** (28)	1993	217	167	207	199	63	173	168	177	110	NR	NR	NR	27	NR	170	NR	NR	NR	NR	NR

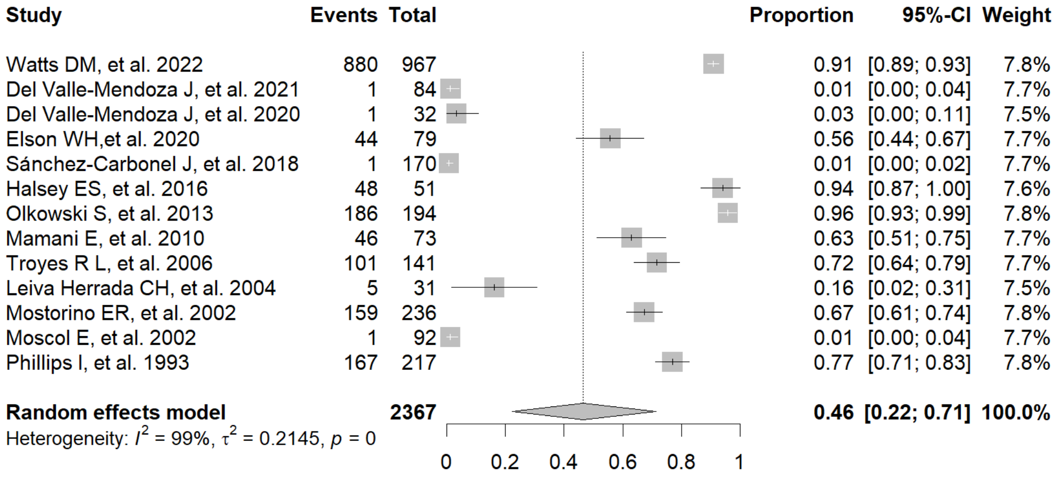

a. Prevalence of chills in Peruvian patients with dengue.

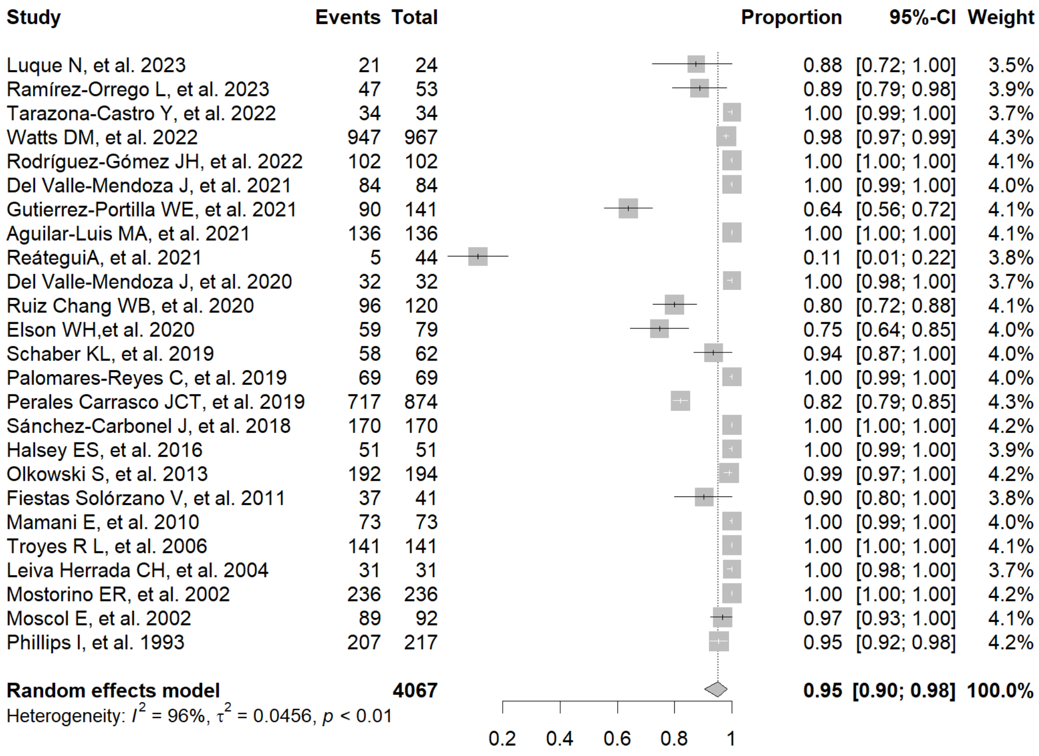

b. Prevalence of fever in Peruvian patients with dengue.

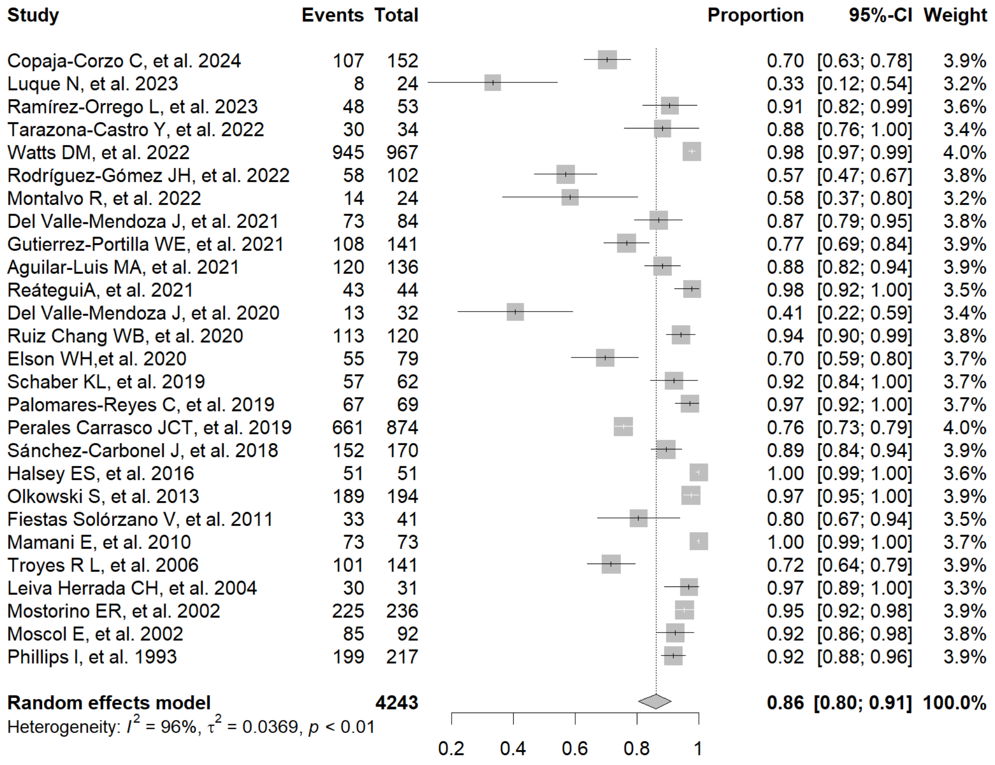

c. Prevalence of headache in Peruvian patients with dengue.

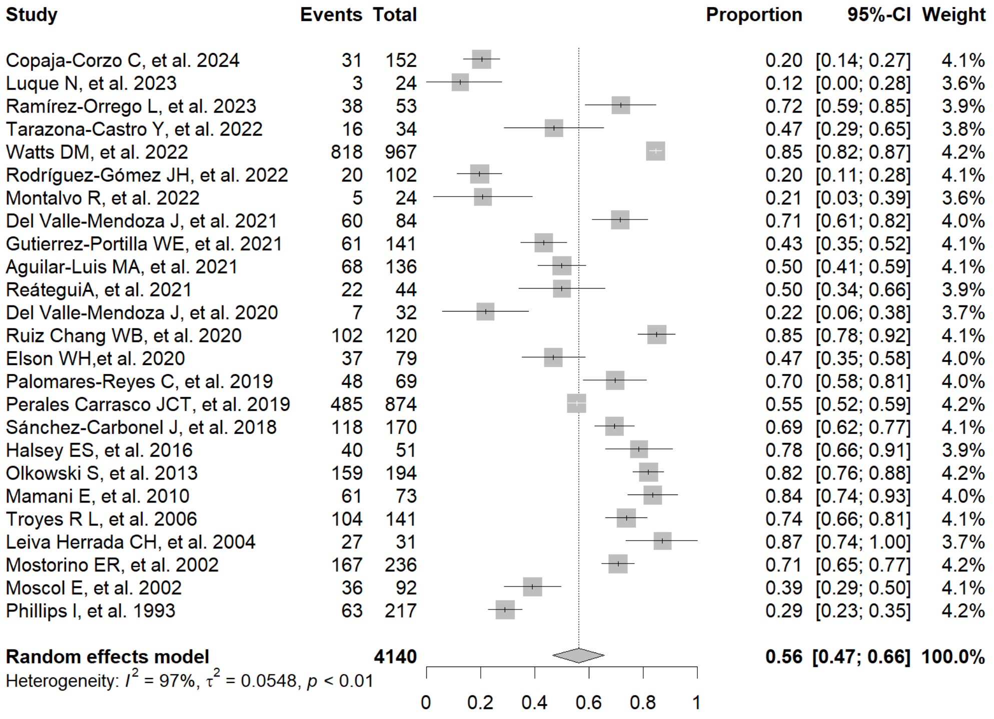

d. Prevalence of retro-orbital pain in Peruvian patients with dengue.

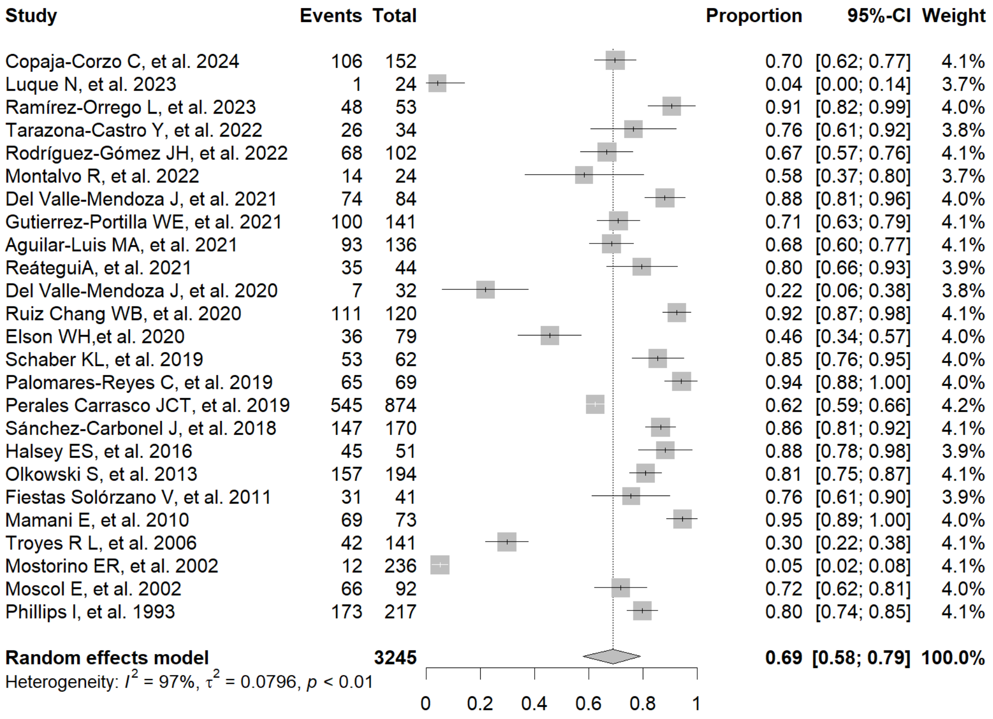

e. Prevalence of myalgia in Peruvian patients with dengue.

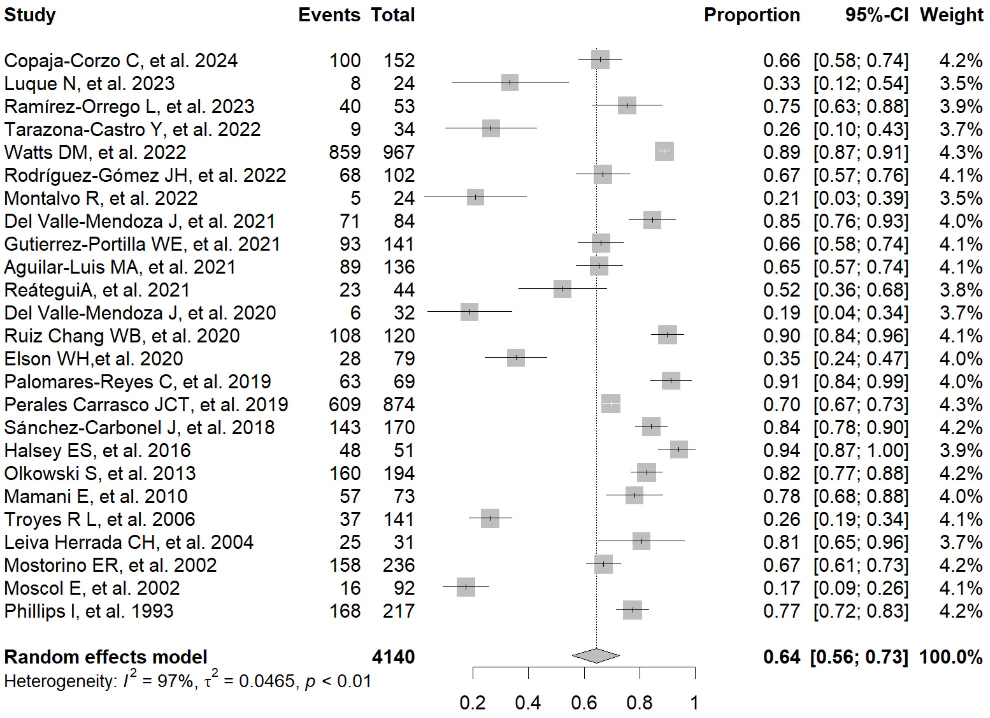

f. Prevalence of arthralgia in Peruvian patients with dengue.

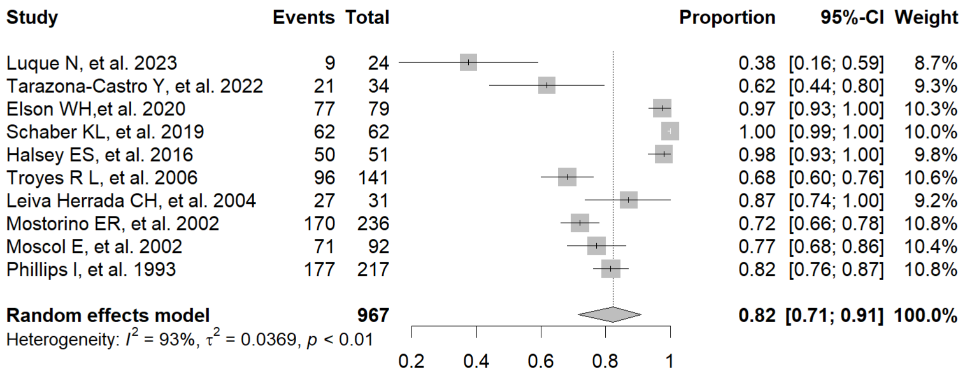

g. Prevalence of general malaise in Peruvian patients with dengue.

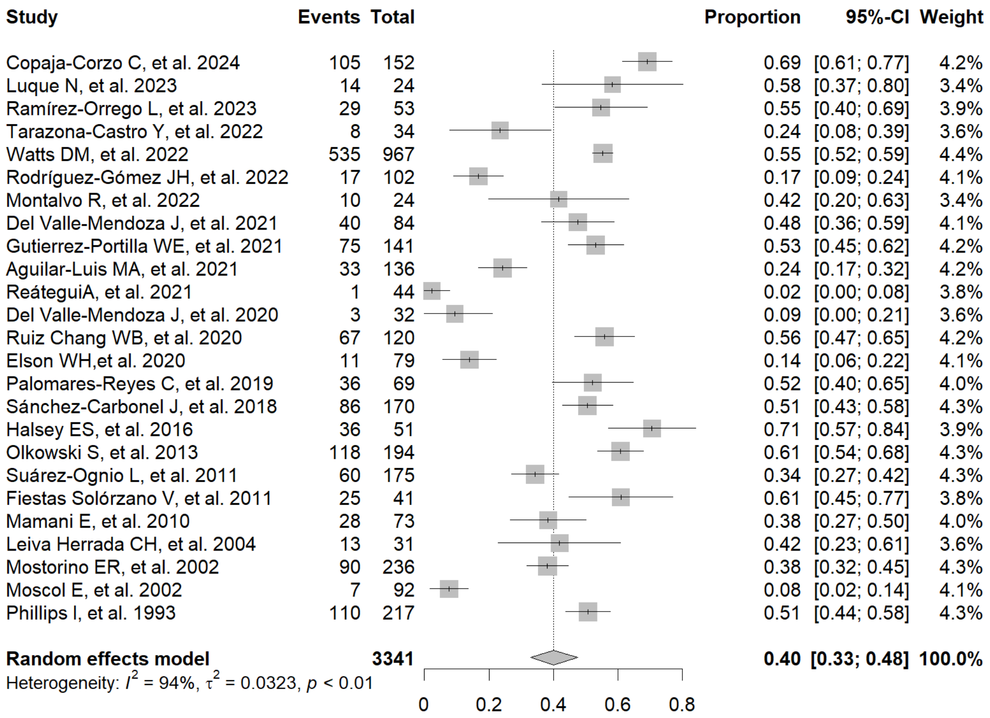

h. Prevalence of nausea/vomiting in Peruvian patients with dengue.

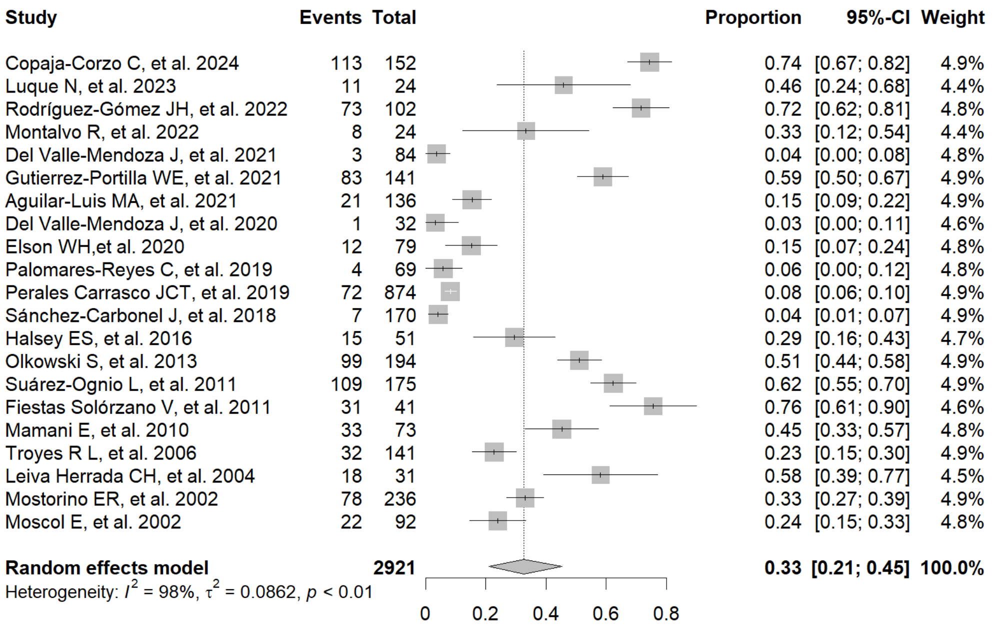

i. Prevalence of abdominal pain in Peruvian patients with dengue.

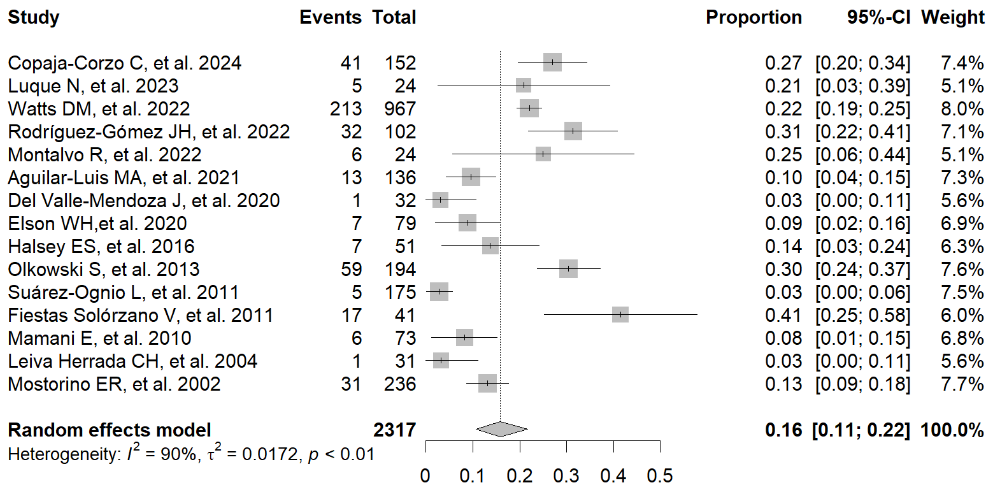

j. Prevalence of diarrhea in Peruvian patients with dengue.

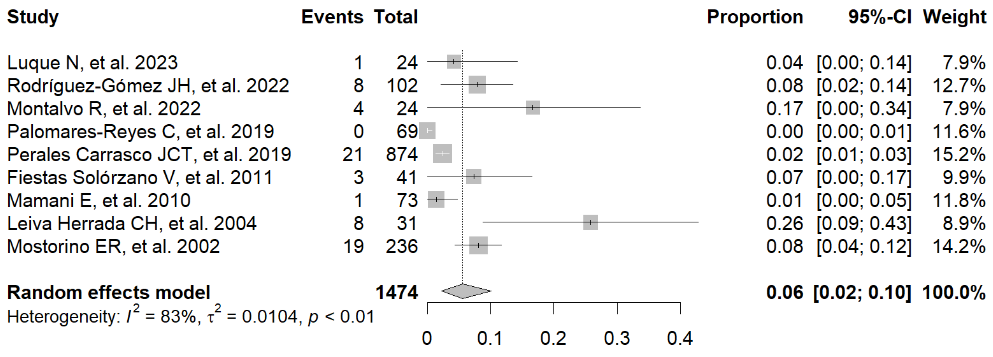

k. Prevalence of hematemesis in Peruvian patients with dengue.

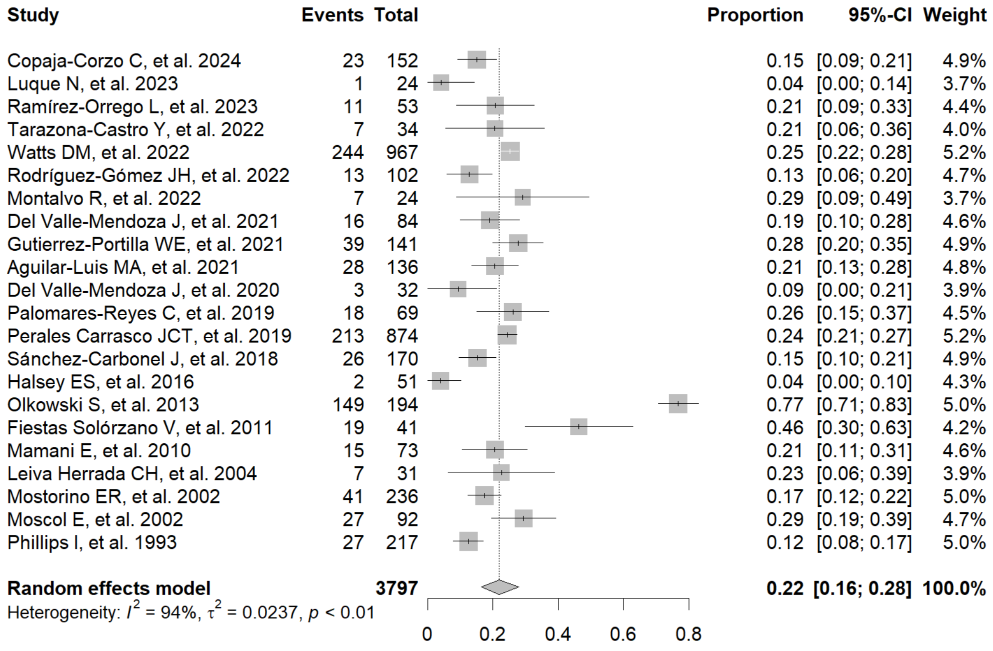

l. Prevalence of rash in Peruvian patients with dengue.

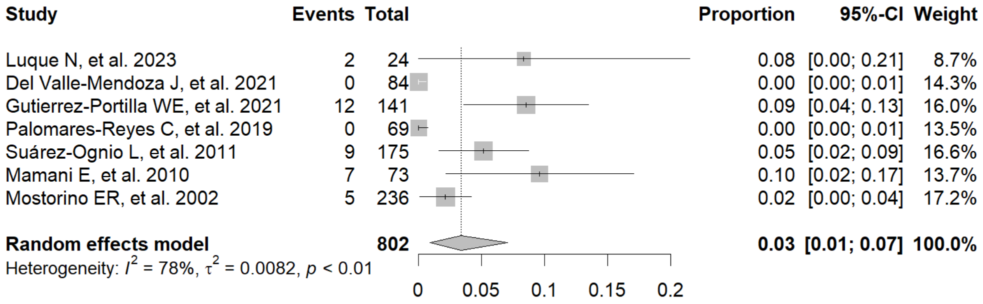

m. Prevalence of jaundice in Peruvian patients with dengue.

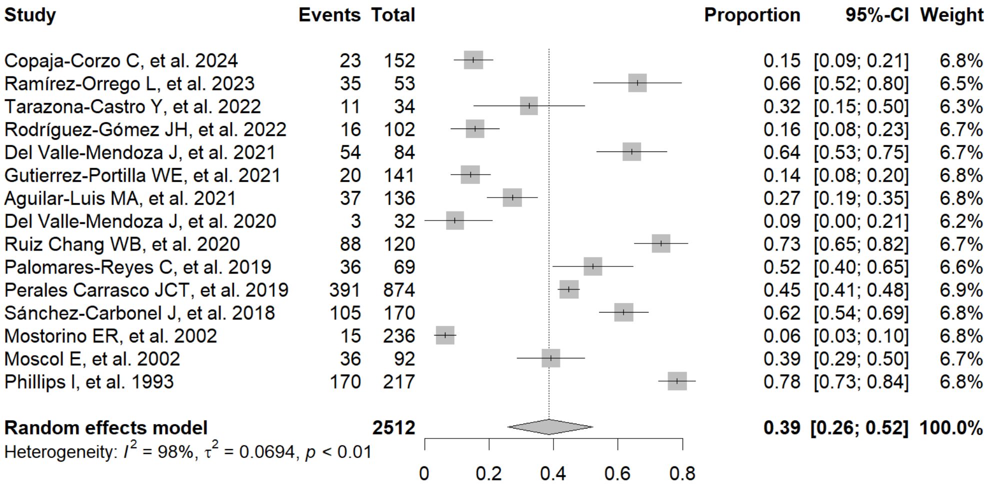

n. Prevalence of low back pain in Peruvian patients with dengue.

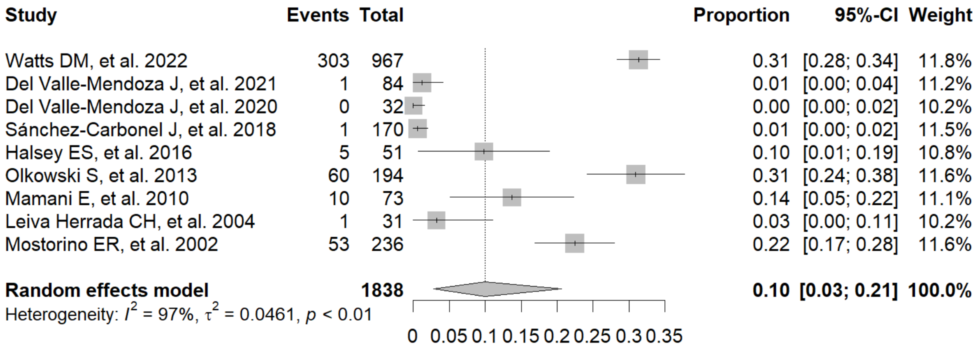

o. Prevalence of cough in Peruvian patients with dengue.

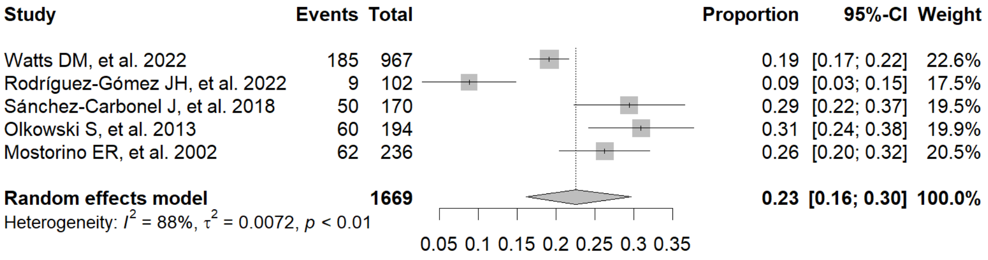

p. Prevalence of sore throat in Peruvian patients with dengue.

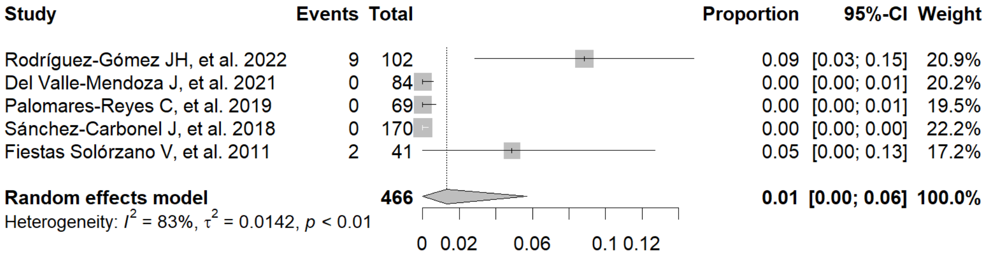

q. Prevalence of melena in Peruvian patients with dengue.

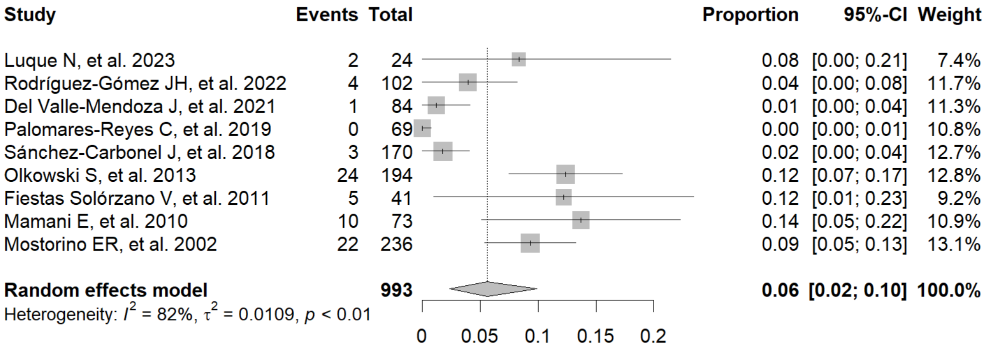

r. Prevalence of petechiae in Peruvian patients with dengue.

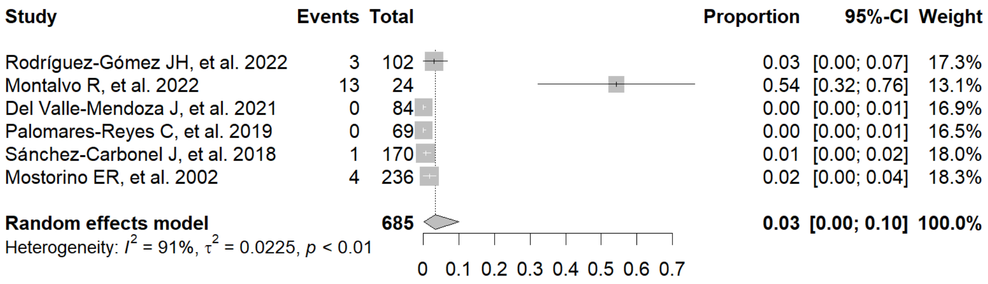

s. Prevalence of ecchymosis in Peruvian patients with dengue.

S1 Figure: Forest diagram illustrating the combined prevalence of clinical manifestations in Peruvian patients with dengue.

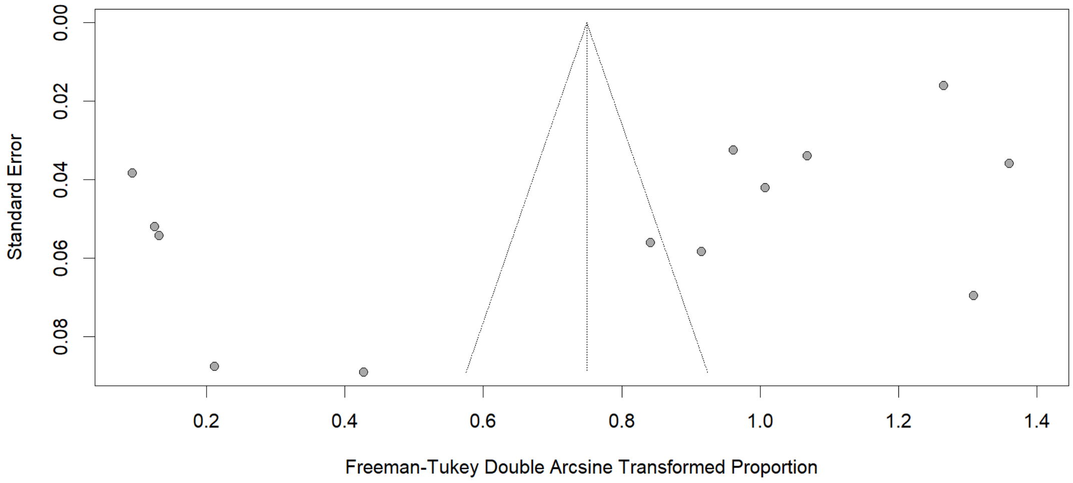

Egger's Test: t = -2.50, df = 11, p-value = 0.0294

a. Studies evaluating the prevalence of chills in Peruvian patients with dengue.

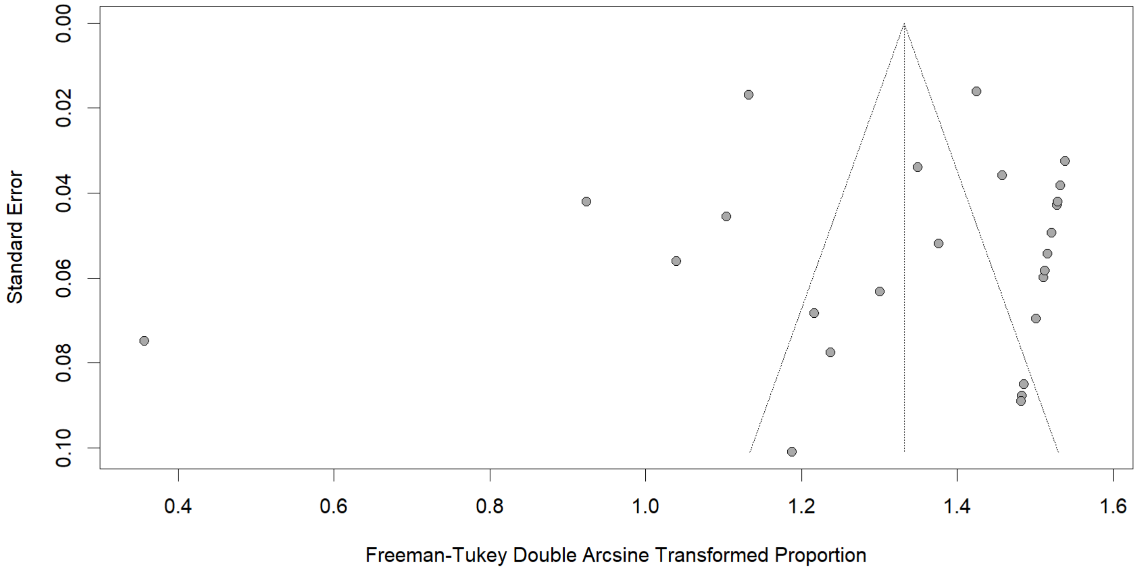

Egger's Test: t = 0.19, df = 23, p-value = 0.8505

b. Studies evaluating the prevalence of fever in Peruvian patients with dengue.

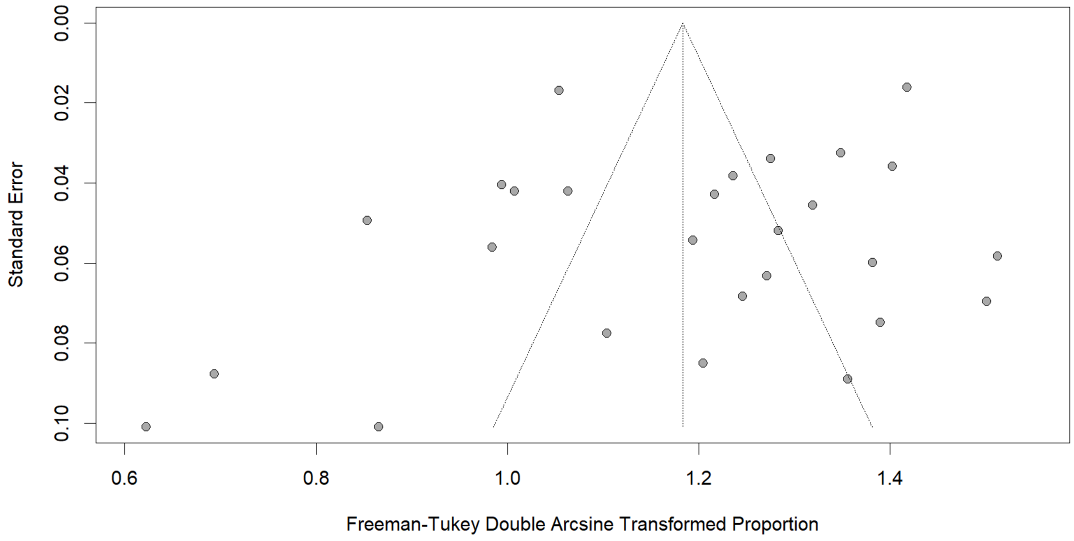

Egger's Test: t = -1.00, df = 25, p-value = 0.3265

c. Studies evaluating the prevalence of headache in Peruvian patients with dengue.

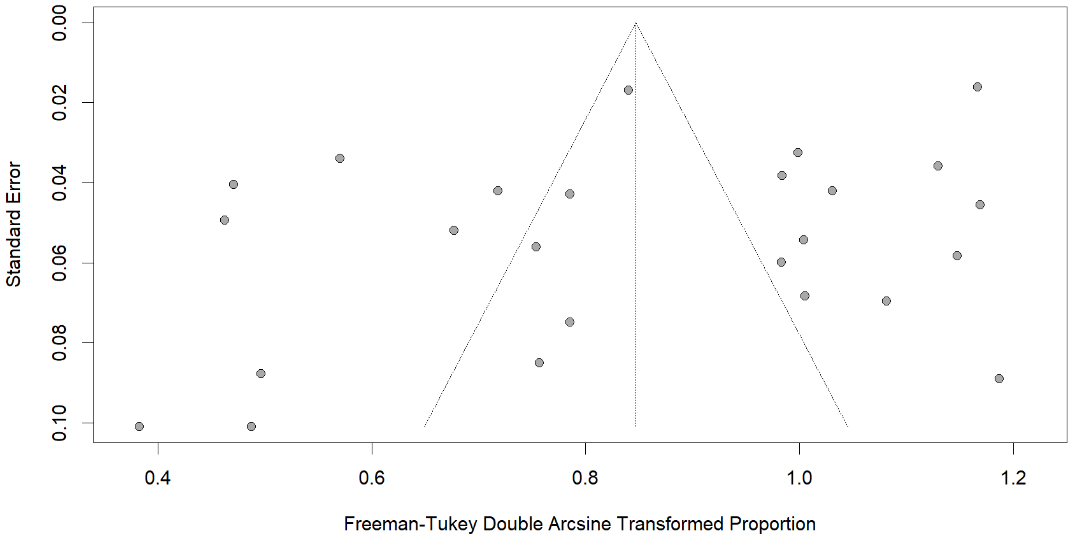

Egger's Test: t = -1.72, df = 23, p-value = 0.0980

d. Studies evaluating the prevalence of retro-orbital pain in Peruvian patients with dengue.

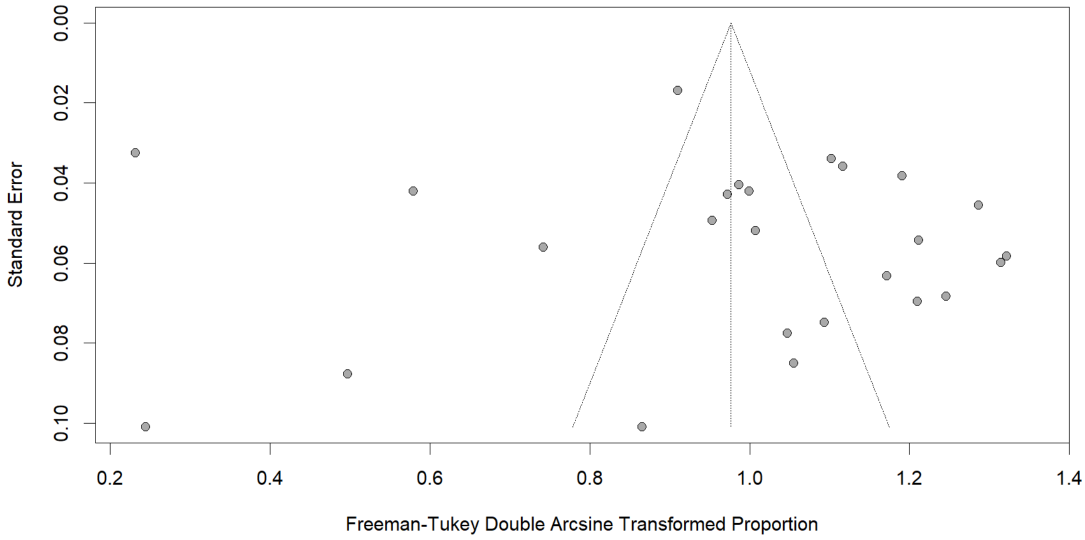

Egger's Test: t = 0.80, df = 23, p-value = 0.4298

e. Studies evaluating the prevalence of myalgia in Peruvian patients with dengue.

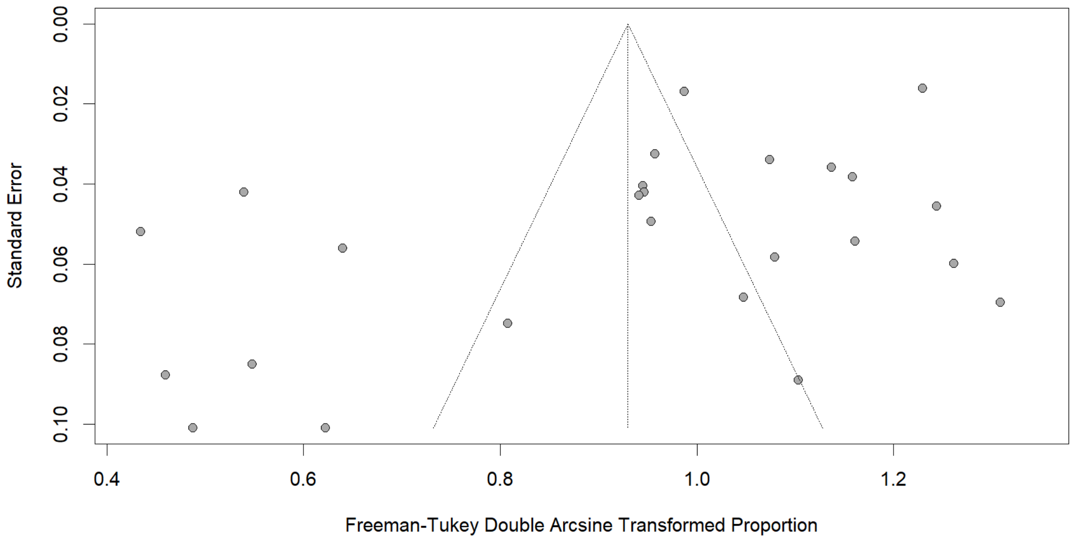

Egger's Test: t = -2.41, df = 23, p-value = 0.0242

f. Studies evaluating the prevalence of arthralgia in Peruvian patients with dengue.

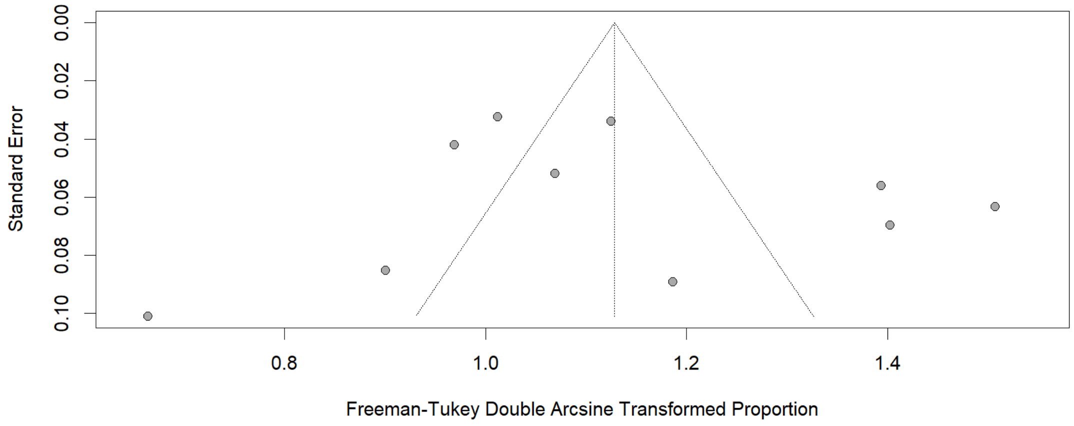

Egger's Test: t = 0.50, df = 8, p-value = 0.6333

g. Studies evaluating the prevalence of general malaise in Peruvian patients with dengue.

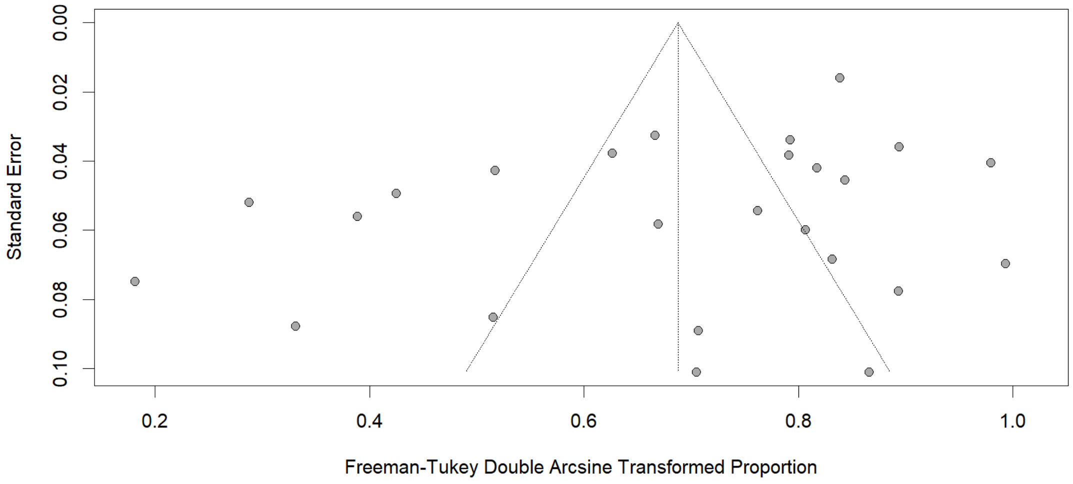

Egger's Test: t = -2.04, df = 23, p-value = 0.0530

h. Studies evaluating the prevalence of nausea/vomiting in Peruvian patients with dengue.

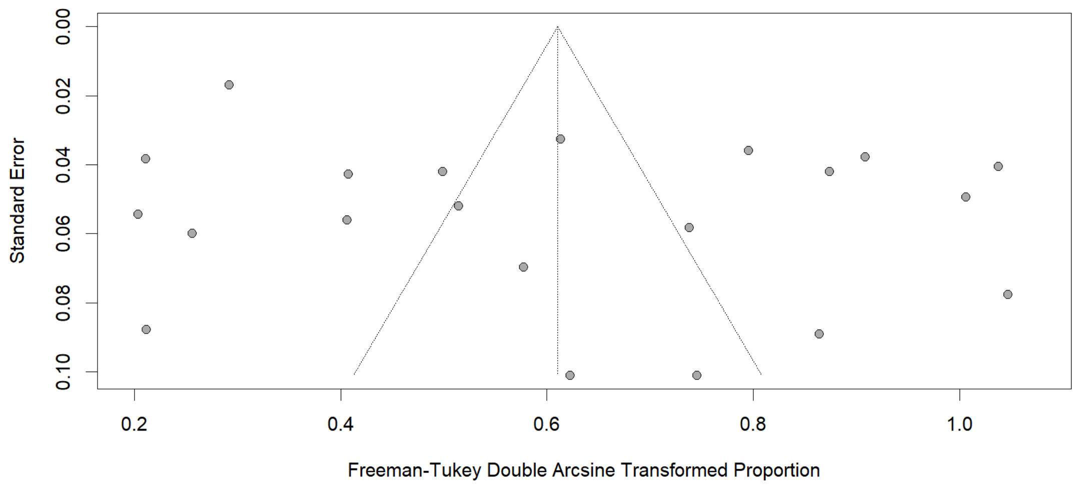

Egger's Test: t = 1.74, df = 19, p-value = 0.0977

i. Studies evaluating the prevalence of abdominal pain in Peruvian patients with dengue.

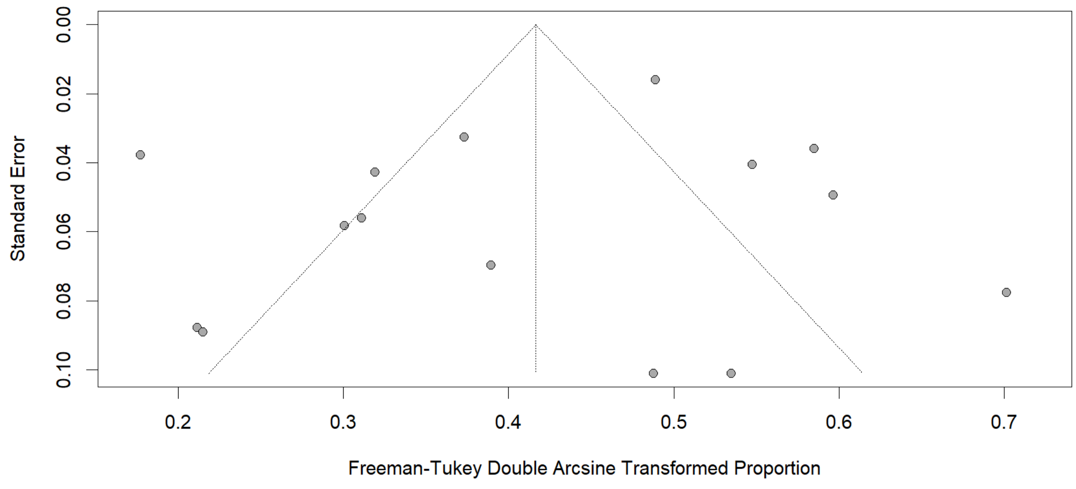

Egger's Test: t = -0.87, df = 13, p-value = 0.3986

j. Studies evaluating the prevalence of diarrhea in Peruvian patients with dengue.

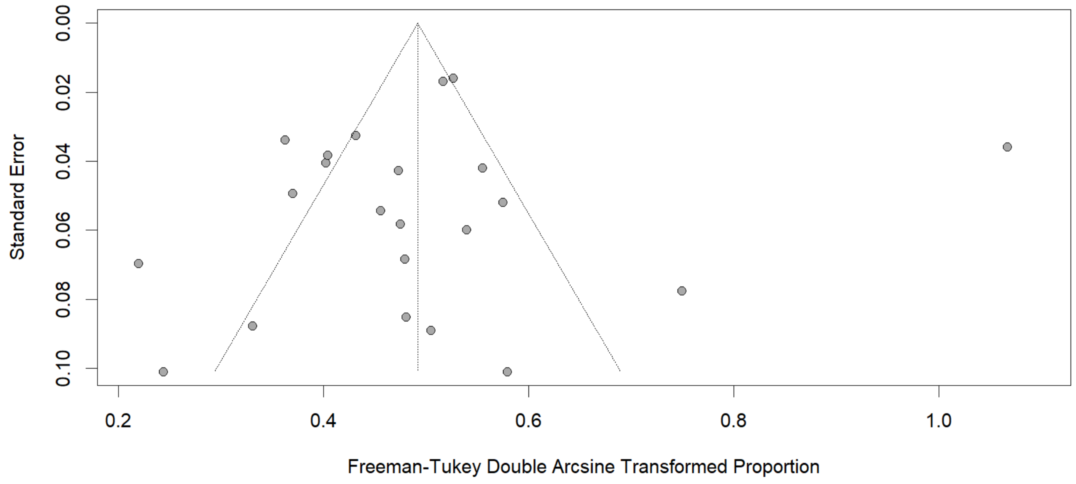

Egger's Test: t = -0.56, df = 20, p-value = 0.5834

k. Studies evaluating the prevalence of rash in Peruvian patients with dengue.

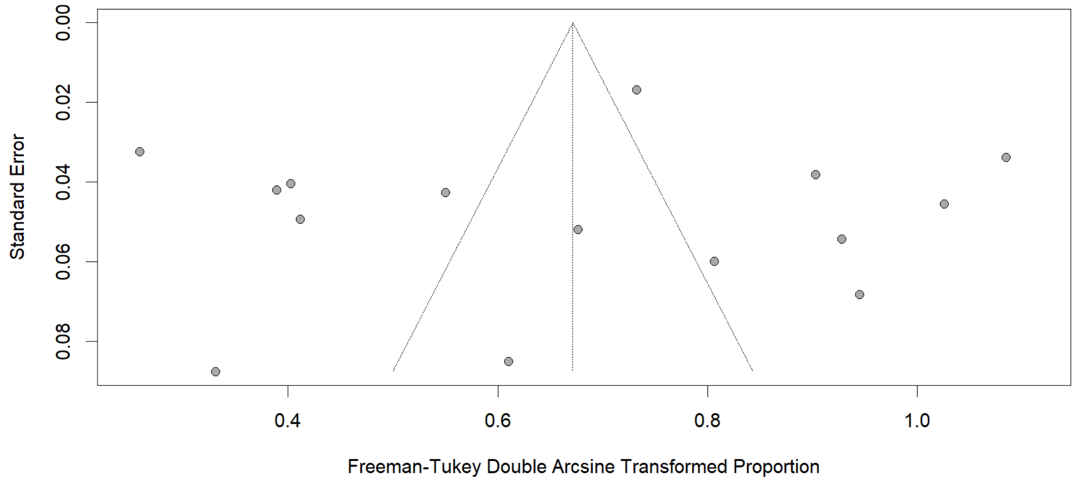

Egger's Test: t = -0.28, df = 13, p-value = 0.7819

l. Studies evaluating the prevalence of low back pain in Peruvian patients with dengue.

S2 Figure: Funnel plot and Egger's test illustrate the publication bias of the included studies.

Dengue without warning signs - DNWS	Dengue with warning signs - DWWS	Severe dengue - SD
Person who lives or has traveled to areas with dengue transmission in the last 14 days and presents fever, usually of 2 to 7 days duration, and at least 2 of the following criteria:<br>1. Nausea/ vomiting<br>2. Exanthema<br>3. Headache/ retro-orbital pain<br>4. Myalgia and arthralgia<br>5. Petechiae or positive tourniquet test<br>6. Leukopenia<br>Cases also include any child coming from or living in an area with dengue transmission, with acute febrile illness, usually of 2 to 7 days and no apparent focus.	Every dengue case that, near and preferably at defervescence, presents one or more of the following signs:<br>1. Intense abdominal pain or tenderness<br>2. Persistent vomiting<br>3. Fluid accumulation (ascites, pleural effusion, pericardial effusion)<br>4. Mucosal bleed<br>5. Lethargy/restlessness<br>6. Postural hypotension (lipothymia)<br>7. Liver enlargement >2 cm<br>8. Progressive increase in hematocrit	Every dengue case that has one or more of the following manifestations:<br>1. Shock or respiratory distress due to severe plasma leakage. Shock evidenced by: weak or undetectable pulse, tachycardia, cold extremities, and capillary perfusion >2 seconds, pulse pressure ≤ 20 mmHg: hypotension in late phase.<br>2. Severe bleeding: based on evaluation by the attending physician (e.g. hematemesis, melena, ample metrorrhagia, central nervous system [CNS] bleeding)<br>3. Severe organ involvement, such as liver impairment (AST or ALT ≥1000 IU), CNS (impaired mental state), heart (myocarditis), or other organs

Requiring strict observation and medical intervention

S3 Figure. Classification of the severity of dengue according to the Pan American Health Organization.

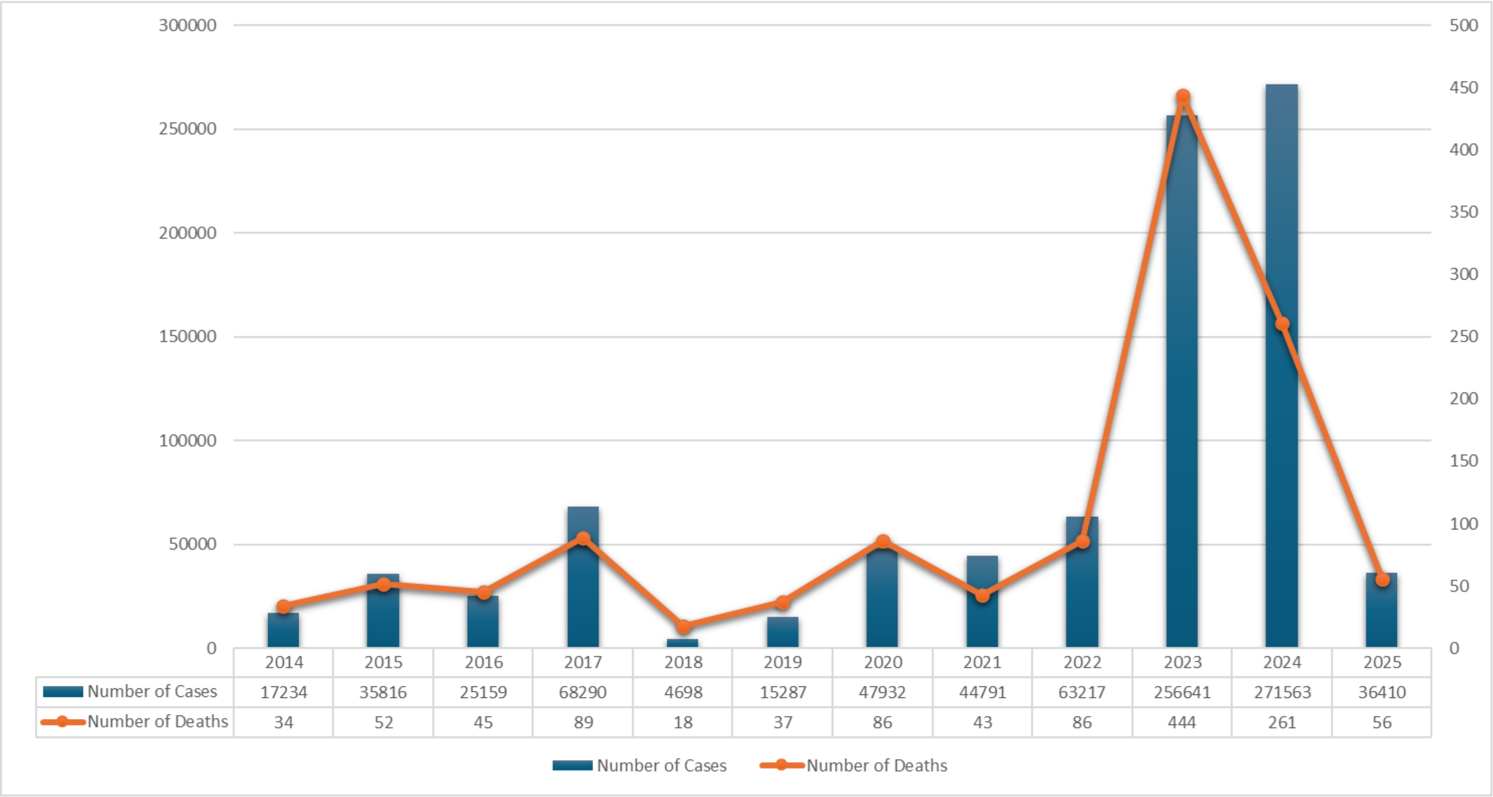

S4 Figure. Dengue cases in Peru during 2014–2025 (week 52) were reported by the National Center for Epidemiology, Prevention, and Disease Control of Peru.
